# Sub-stoichiometric 2D covalent organic frameworks from tri- and tetratopic linkers

**DOI:** 10.1038/s41467-019-10574-6

**Published:** 2019-06-19

**Authors:** Tanmay Banerjee, Frederik Haase, Stefan Trenker, Bishnu P. Biswal, Gökcen Savasci, Viola Duppel, Igor Moudrakovski, Christian Ochsenfeld, Bettina V. Lotsch

**Affiliations:** 1 Max Planck Institute for Solid State Research, Heisenbergstraße 1, 70569 Stuttgart, Germany; 2 Department of Chemistry, University of Munich (LMU), Butenandtstraße 5-13, 81377 München, Germany; 3 Cluster of Excellence e-conversion, Schellingstraße 4, 80799 München, Germany; 4 Center for Nanoscience, Schellingstraße 4, 80799 München, Germany; 5 Present Address: Institute for Integrated Cell-Material Sciences (WPI-iCeMS), Kyoto University, Kyoto, 606-8501 Japan

**Keywords:** Polymers, Porous materials, Polymers

## Abstract

Covalent organic frameworks (COFs) are typically designed by breaking down the desired network into feasible building blocks - either simple and highly symmetric, or more convoluted and thus less symmetric. The linkers are chosen complementary to each other such that an extended, fully condensed network structure can form. We show not only an exception, but a design principle that allows breaking free of such design rules. We show that tri- and tetratopic linkers can be combined to form imine-linked [4 + 3] sub-stoichiometric 2D COFs featuring an unexpected *bex* net topology, and with periodic uncondensed amine functionalities which enhance CO_2_ adsorption, can be derivatized in a subsequent reaction, and can also act as organocatalysts. We further extend this class of nets by including a ditopic linker to form [4 + 3 + 2] COFs. The results open up possibilities towards a new class of sub-valent COFs with unique structural, topological and compositional complexities for diverse applications.

## Abstract (web summary)

Using complementary linkers as building blocks in the design of 2D covalent organic frameworks (COFs) limits the formation of compositionally and structurally complex networks. Here, the authors demonstrate a COF with a *bex* topology by combining non-complementary triangular and rectangular linkers.

## Introduction

**Fig. 1:**
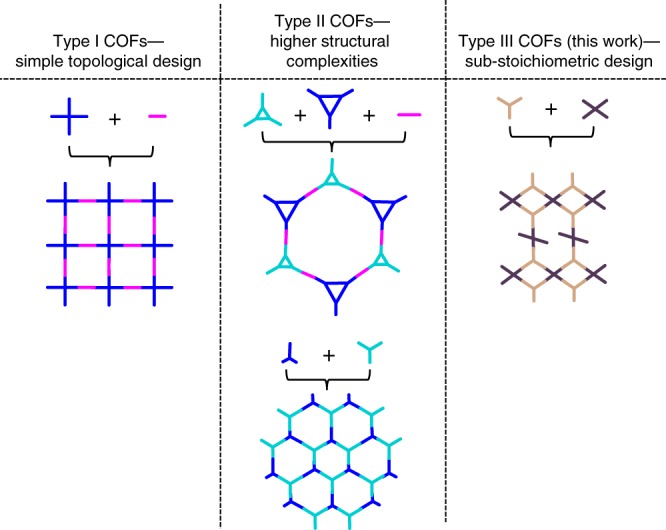
Hierarchy of the evolution of structural complexity in COFs

Covalent organic frameworks (COFs) represent an intriguing class of crystalline porous materials, where appropriately functionalized organic molecules are linked with an atomic level precision to give extended periodic structures^1–6^. Such materials are synthesized by topologically directed condensation reactions, based on matching the geometry of the building blocks to form the desired net composed of the respective vertex and edge linker units^3,7–9^. The shapes and dimensions of the vertex and the edge linkers determine the pore shape and size, respectively. Most of the 2D COF structures reported till date are based on four simple topologies for which the vertices are connected by only one kind of edge—the honeycomb, square, kagome, and hexagonal lattice complexes^3^. In order to make the COFs, these nets are typically deconstructed into highly symmetric building blocks (e.g., Type I COFs, Fig. 1). On the other hand, increased compositional and structural complexity is paramount in order to broaden the scope and applicability of COFs (or any other material). Reports in this regard are limited to either using complex molecules or macromolecules as vertices^10–20^, or using combinations of linear linkers of different lengths with appropriate vertex molecules^21,22^, to arrive at complicated network structures (e.g., Type II COFs, Fig. 1). However, most such efforts produce deformations of the aforementioned simple topologies only. More importantly, most of these approaches involve cumbersome and time consuming multistep organic synthesis before the COF formation reaction which is yet to be understood well and controlled. In all the possibilities discussed thus far, the vertices and the linkers are always complementarily chosen such that formation of a fully condensed network structure is possible. In other words, only fully condensed structures are considered as targets for synthesis of COFs. Synthesis is not attempted if due to geometric constraints or otherwise this rule is deemed not possible to be followed, thus limiting creativity.

Contrary to these typical design principles, in this manuscript, we show that triangular tritopic and rectangular tetratopic linkers can actually be combined to form a crystalline, dual pore 2D COF belonging to a previously unidentified *bex* topology with two distinct vertex molecules (Fig. 1, Type-III COFs). Uniquely, in such COFs the tetratopic linker simultaneously acts as a bi- and tetradentate linker in two different coordination environments and thus inherently results in uncondensed functional groups in the network. This has significant implications with regard to the potential of COFs for applications in diverse fields, including sensing^23,24^, storage^25,26^, (photo)catalysis^27–29^, ion conduction^30,31^, and optoelectronics^32,33^. Such diverse applicability demands diversified structural characteristics and importantly, appropriate functionality, which is often difficult to achieve in a single-step and requires post-synthetic modification of the framework. With examples limited to only a few^5,26,34,35^, it is still challenging to tune the structure and the physical properties of the COF post-synthetically while maintaining its crystallinity and porosity. This is because generating functional groups in the framework by post-synthetic modification requires orthogonal reaction chemistry, which also limits probable options considerably. In this regard, the unconventional combination of building blocks to produce periodic, uncondensed amine functional groups in the fully extended, crystalline and porous imine-linked COFs, is a new approach toward parallel and simultaneous functionalization of 2D COFs. Also, this method of introducing free functional groups by generating defined sub-valent COFs is fundamentally different from previous examples that rely on the introduction of defects^36,37^, or the introduction of periodic linker vacancies into a saturated host lattice to introduce dangling functional groups in the framework as reported by Loh and co-workers^38^. We further show that the free amine functional groups enhance CO_2_ adsorption of the framework, can be transformed in a subsequent reaction, and can also act as catalysts in organic transformations.

All three-component COFs reported thus far use building blocks of only two different symmetries^13,21,22,39^. In this report, we also demonstrate the combination of tritopic, tetratopic, and linear ditopic linkers to form COFs, which have crystallinity and surface area comparable to the state-of-the art examples.

This class of sub-stoichiometric COFs with a frustrated bonding network prompts us to think outside the set rules of topological deconstruction and paves way for a new class of structures with unique structural, topological, and compositional complexities. Such COFs are inherently linked to free functional groups, and can be exploited in a diverse, application-specific manner.

## Results

### Synthesis of PT- and PY-COFs

**Fig. 2:**
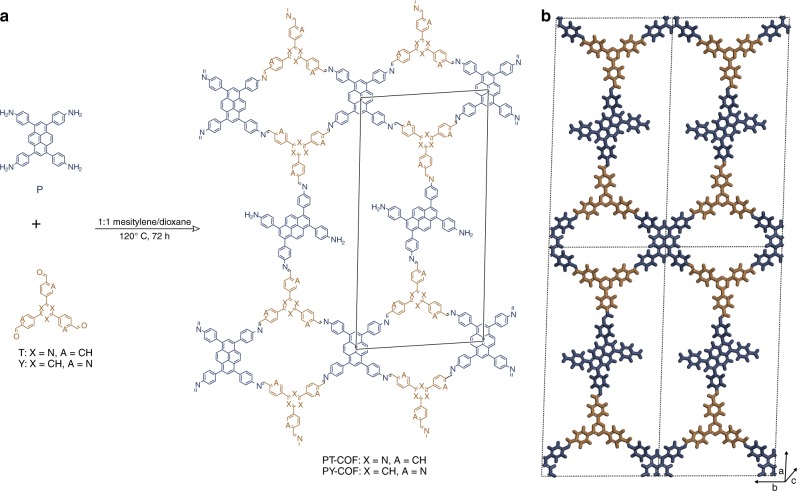
Structure of PT- and PY-COFs. **a** Synthesis and molecular structure of PT- and PY-COFs. The in-plane unit cell is outlined in black. **b** Illustration of the COF structure model with *bex* toplogy (viewed perpendicular to the *ab* plane)

Triazine tribenzaldehyde (T) and benzene tripicolinaldehyde (Y) were chosen as the triangular tritopic linkers for reaction with pyrene tetraaniline (P) as the tetratopic linker. A fully condensed 2D framework cannot be envisaged from a combination of molecules with such symmetries, and thus periodic, crystalline frameworks are typically not expected to form. Nevertheless, when these linkers are reacted in an equimolar ratio in 1:1 mesitylene/dioxane under solvothermal conditions, crystalline PT-, and PY-COFs result (Fig. 2 and Supplementary Methods). Other typical solvent combinations and different ratios of mesitylene and dioxane, as well as different molar ratios of the reactants, either had no noticeable effect or decreased crystallinity while still retaining the powder x-ray diffraction (PXRD) pattern (Supplementary Methods).

### IR and NMR characterization of PT- and PY-COFs

**Fig. 3:**
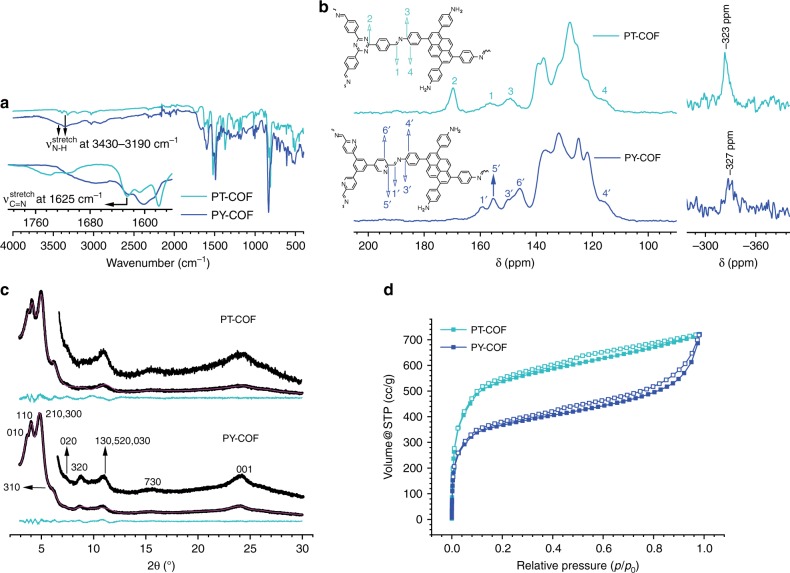
Characterization of PT- and PY-COFs. **a** FT-IR characterization of PT- and PY-COFs. Free amine functionalities can be identified by the characteristic *ν*_N-H(stretch)_ in the region 3430 cm^−1^–3190 cm^−1^. The inset shows the presence of *ν*_C=N(stretch)._ **b** ^13^C ssNMR spectra with corresponding assignments. The unassigned signals correspond to the pyrene moiety and the phenyl rings. ^15^N ssNMR depicting the free amine functionalities in PT- and PY-COFs is shown on the right as an inset. **c** PXRD patterns of PT- and PY-COFs with corresponding Rietveld refinements (magenta) showing good fits to the experimental data (black) with minimal differences. The turquoise traces show the difference between the experimental data and the refinement. The corresponding Miller indices of the identifiable reflections are shown as well. **d** Argon sorption isotherms of PT- and PY-COFs at 87 K. Filled and open symbols represent the adsorption and the desorption branches, respectively

Fourier transform infrared spectroscopy (FT-IR) measurements (Fig. 3a) of PT- and PY-COFs show almost complete disappearance of *ν*_C=O(stretch)_ at ~1700 cm^−1^ corresponding to the aldehyde groups in the starting materials, T and Y (Supplementary Fig. 1). The disappearance of the carbonyl stretching signal is accompanied by the appearance of *ν*_C=N(stretch)_ at 1625 cm^−1^ corresponding to the imine bonds formed in the framework by condensation of the aldehyde and the amine groups in the starting materials. Interestingly, similar to that in P (Supplementary Fig. 1), *ν*_N-H(stretch)_ in the region 3430 cm^−1^–3190 cm^−1^ is observed for both PT- and PY-COFs. ^13^C cross-polarization magic angle spinning solid-state NMR (ssNMR) spectroscopy (Fig. 3b) shows the disappearance of the aldehyde carbonyl ^13^C resonance at ~190 ppm as present in the precursor aldehydes, T, and Y^27,40,41^. This is accompanied by the appearance of the characteristic imine ^13^C signal^41,42^ at 157 ppm (1) for PT-COF and at 159 ppm (1′) for PY-COF proving the condensation of the starting materials into the framework. Retention of the molecular structure of the building blocks in the framework is also evident from an analysis of the ^13^C NMR spectrum. For PT-COF, the signals for carbon 2, 3, and 4 can be identified at 170 ppm, 150 ppm, and 116 ppm, respectively^42^. Likewise, for PY-COF, carbon 3′, 4′, 5′, and 6′ appear at 150 ppm, 116 ppm, 155 ppm, and 146 ppm, respectively. This analysis is further corroborated with ^15^N-ssNMR measurements (Fig. 3b and Supplementary Figs 2, 3) and corresponding quantum-chemical calculations (Supplementary Fig. 4–9 and Supplementary Tables 1, 2). For PT-COF, the imine nitrogen 7 appears at −46 ppm and the triazine nitrogen 8 appears at −127 ppm^42^. For PY-COF, the imine (7′) and the pyridine (10′) nitrogen appear between −40 ppm and −78 ppm. The signal at −262 ppm in the ^15^N ssNMR spectrum of PY-COF probably arises from oxidation of a fraction of the imine-linkages to amides in the COF (Supplementary Figs 9, 10, Supplementary Table 1)^43–47^. Interestingly, both PT- and PY-COFs show the presence of free amine functional groups (9 and 9′) at −323 ppm to −330 ppm (Fig. 3b, inset).

### PXRD analysis and structural modeling of PT- and PY-COFs

PY- and PT-COFs are crystalline materials (see Supplementary Fig. 11 for chemical stability tests). The experimental PXRD patterns (Fig. 3c) show eight reflections at 3.7, 4.0, 4.9, 6.1, 7.3, 8.7, 10.9, and 15.2° 2*θ*. In addition, a broad stacking reflection can be seen at 2*θ* = 24° for both COFs hinting towards the formation of 2D rather than a 3D-COF (vide infra). The PXRD patterns could be simulated by a structure model (Supplementary Methods) as shown in Fig. 2a and b. In this model, the tetratopic P plays a dual role, a behavior that is currently unknown in COF chemistry. The triangular linkers connect two P molecules at two points on both sides; P thus acts as a node and is tetra-coordinated to produce a ribbon-like architecture. The free –CHO termini of the triangular linkers constituting the ribbons are then interconnected by a single P molecule by its diagonal amine groups, which now acts as a linear ditopic linker. As a consequence, two uncondensed –NH_2_ groups result per unit cell, the presence of which were confirmed with ^15^N-ssNMR and IR experiments. The unit cell parameters for this structure model with *P*1 space group and *bex* crystallographic network topology were obtained by force-field optimizations and were then Pawley refined against the experimental PXRD pattern (Supplementary Table 3). The refined unit cell parameters are observed to be similar to the parameters obtained by structure simulations. The structure model for AA-type stacking yields the best agreement with the experimental pattern (Supplementary Fig. 12). Finally, using this model and the parameters obtained from the Pawley fit, the experimental powder pattern was Rietveld refined, yielding the unit cell parameters of *a* = 52.516 Å, *b* = 23.556 Å, *c* = 3.681 Å, and *α* = *β* = 90°, *γ* = 90.759°, *R*_wp_ = 3.22 for PT-COF (Supplementary Data 1) and *a* = 52.718 Å, *b* *=* 24.117 Å, *c* = 3.815 Å, and *α* = *β* = 90°, *γ* = 88.393°, *R*_wp_ = 2.0 for PY-COF (Supplementary Data 2). It is important to note that the *bex* net in the present case is a more complex net and has a transitivity of 222 with two kinds of vertices from two different molecules as vertices in the network structure and has not been previously identified for COFs^48^. Almost all of the hundreds of 2D COF structures with considerable complexities produced till date belong to only four highly symmetric network topologies with minimal transitivities^3^. A 3D-COF based on the *tbo* net, as known from the metal organic framework HKUST-1^49^, can also be envisaged by connecting the P and T/Y linkers and was thus modeled (Supplementary Fig. 13, 14). However, the experimental PXRD patterns, with the stacking reflection at 2*θ* = 24°, did not match with that expected for this model. It should be noted that the formation of a crystalline 2D COF from P and T/Y linkers is classically inconceivable because these linkers cannot form a fully condensed 2D framework. It is also worth noting the reported formation of 3D-COFs from tetraphenylethylene-based tetratopic and triphenylbenzene/triphenylamine-based tritopic linkers^50^. This points to a probable design principle for the formation of such 2D sub-stoichiometric COFs: the increased planarity of linkers (as with our P and T/Y linkers) possibly increases the predilection towards stacking and thus the consequent formation of such structures over stoichiometric 3D-COFs.

### Synthesis of PT_2_B- and PY_2_B-COFs

**Fig. 4:**
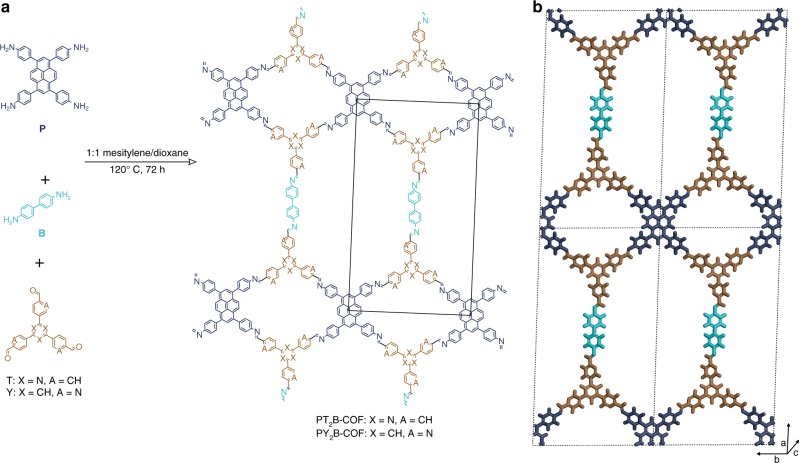
Structure of PT_2_B- and PY_2_B-COFs. **a** Synthesis and molecular structure of PT_2_B- and PY_2_B-COFs. The in-plane unit cell is outlined in black. **b** Illustration of the COF structure model (viewed perpendicular to the *ab* plane)

In order to further validate the structure model, we decided to decouple the two linking modes of P and check if a COF could be made by replacing one equivalent of this linker with the linear ditopic benzidine (B) linker. In the resulting structure, the ribbons should now be linked by B molecules instead of P, and thus the COF should not have any free-amine functional groups. Thus, P, T/Y, and B were reacted in a 1:2:1 molar ratio in the same solvent mixture as before under the same conditions to get highly crystalline PT_2_B- and PY_2_B-COFs (Fig. 4 and Supplementary Methods).

### IR and NMR characterization of PT_2_B- and PY_2_B-COFs

**Fig. 5:**
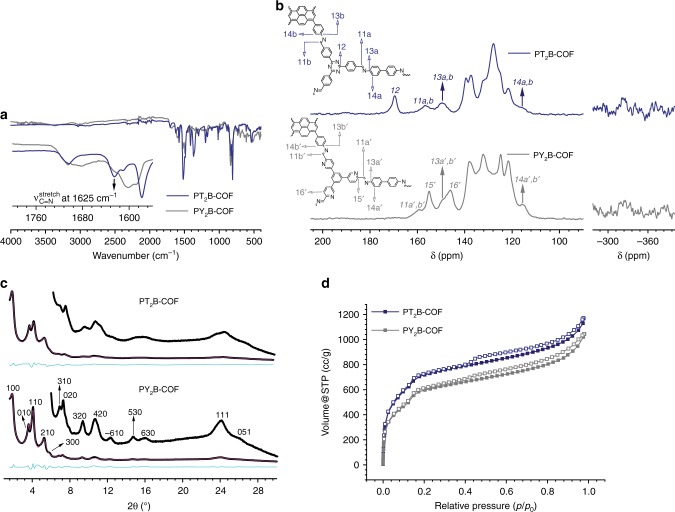
Characterization of PT_2_B- and PY_2_B-COFs. **a** FT-IR characterization of PT_2_B- and PY_2_B-COFs. The inset shows the presence of *ν*_C=N(stretch)._ **b** ^13^C ssNMR spectra with corresponding assignments. The unassigned signals correspond to the pyrene moiety and the phenyl rings. ^15^N ssNMR depicting the absence of free amine functionalities in PT_2_B- and PY_2_B-COFs is shown on the right as an inset. **c** PXRD patterns of PT_2_B- and PY_2_B-COFs with corresponding Rietveld refinements (magenta) showing good fits to the experimental data (black) with minimal differences. The turquiose traces show the difference between the experimental data and the refinement. The corresponding Miller indices of the identifiable reflections are shown as well. **d** Argon sorption isotherms of PT_2_B- and PY_2_B-COFs at 87 K. Filled and open symbols represent the adsorption and the desorption branches, respectively

FT-IR measurements (Fig. 5a) again show a near complete loss of *ν*_C=O(stretch)_ signal corresponding to the aldehyde groups in the starting materials and a simultaneous appearance of *ν*_C=N(stretch)_ at 1625 cm^−1^, validating the formation of the framework. Indeed, as expected, the *ν*_N-H(stretch)_ signal, as observed for PT- and PY-COFs, is absent in PT_2_B- and PY_2_B-COFs. ^13^C ssNMR spectrum (Fig. 5b) of PT_2_B- and PY_2_B-COF is very similar to PT- and PY-COF, respectively, and again shows the disappearance of the aldehyde carbonyl ^13^C resonance at ~190 ppm and appearance of an imine ^13^C signal at 157 ppm (11) for PT_2_B-COF and at 159 ppm (11′) for PY_2_B-COF proving the formation of the framework. Retention of the molecular structure of the building blocks in the framework is again evident. ^15^N ssNMR is identical to PT- and PY-COFs (Supplementary Fig. 15), except for an almost complete absence of nitrogen signals corresponding to free amine functional groups (Fig. 5b, inset).

### PXRD analysis and structural modeling of PT_2_B- and PY_2_B-COFs

PT_2_B- and PY_2_B-COFs have higher crystallinity as compared with PT- and PY-COFs. The PXRD patterns (Fig. 5c) have 12 and 14 prominent reflections, respectively, for PT_2_B- and PY_2_B-COF at 1.8, 3.6, 4.1, 5.3, 5.9 (for PY_2_B-COF), 6.9, 7.3, 9.4, 10.6, 12.3, 14.7 (for PY_2_B-COF), 15.9, 24.2, 25.8–26.5° 2*θ*. The Pawley refined unit cell parameters (Supplementary Table 3) are again similar to the parameters simulated by modeling the structure (Supplementary Fig. 16). The unit cell parameters as obtained by Rietveld refinement of the experimental powder pattern were *a* = 46.264 Å, *b* = 23.872 Å, *c* = 3.802 Å, and *α* = *β* = 90°, *γ* = 90.247^o^, *R*_wp_ = 2.64 for PT_2_B-COF (Supplementary Data 3) and *a* = 45.845 Å, *b* = 24.352 Å, *c* = 3.743 Å, and *α* = *β* = 90°, *γ* = 90.981°, *R*_wp_ = 2.59 for PY_2_B-COF (Supplementary Data 4). The replacement of the longer P in PT- and PY-COFs with a shorter B in PT_2_B- and PY_2_B-COFs to interlink the ribbons is immediately apparent from the crystal parameters with the corresponding unit cell length *a* decreasing from 52.5–52.7 Å in the former to 46.2–45.8 Å in the latter. In addition, the unit cell length *b*, corresponding to the separation of the pyrene moieties in the ribbon in both the COF systems stays the same at ~23.5–24.4 Å. This further proves the correctness of our structural model. The lower crystallinity of PT- and PY-COFs compared with PT_2_B- and PY_2_B-COFs could be argued to be owing to the presence of free amine groups in the P molecule that acts as the linker interconnecting the ribbons. Reaction only along the diagonal amine groups of such linkers will give rise to the described structure; condensation and reaction propagation along other directions will lead to defects and disorder reducing the crystallinity and crystallite size as observed with TEM measurements (vide infra). Making COFs by combining three different linkers has been reported, albeit using intuitive combinations of either two differently sized linear linkers of the same geometry^13,21,22^, or using two molecules of similar geometry but having different linking groups^39^. PT_2_B- and PY_2_B-COFs, on the other hand, are the first example of COFs made from a combination of three linkers with different topicity.

### TEM analysis

**Fig. 6:**
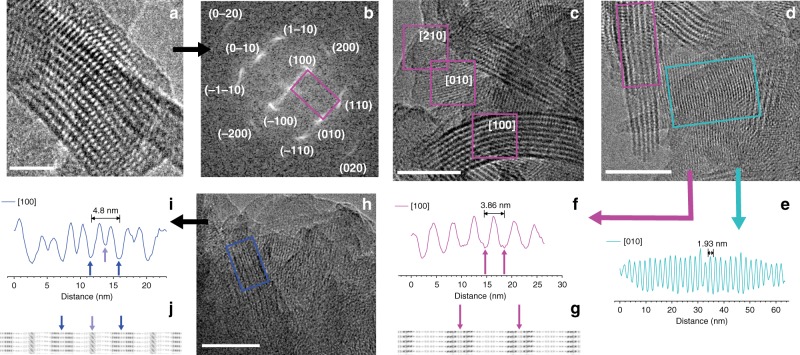
TEM analysis. **a** A PY_2_B-COF crystallite viewed along [001] showing the pores in the structure. **b** Fast Fourier Transform (FFT) filtered image of **a** showing the different planes in the PY_2_B-COF crystallite in accordance with the PXRD data. **c** Individual crystallites of PY_2_B-COF in different orientations. Multiple lattice spacings can be identified as shown. **d** Individual crystallites of PT_2_B-COF in different orientations. **e** Line scan analysis in the [010] direction corresponding to the turquoise outlined region in **d**. A small *d*-spacing of 1.93 nm can be identified and is similar for all four COFs showing a similar constitution of the ribbon structure. A line scan analysis with monomodal intensity variation in the [100] direction (magenta outline) is shown in **f**. The magenta arrows correspond to the electron rich ribbons in PT_2_B-COF as shown with magenta arrows in the projected potential map **g** of the COF. Dark contrast corresponds to higher projected potentials. **h** Individual crystallites of PT-COF in different orientations. Multiple lattice spacings can be identified. A line scan analysis in the [100] direction (blue outline) showing bimodal intensity variation is shown in **i**. The deep blue arrows correspond to the electron rich ribbons in PT-COF as shown with similar arrows in the projected potential map **j** of the COF, wherein dark contrast corresponds to higher projected potentials. The light blue arrow in the line scan analysis designates the bi-coordinating P linkers in PT-COF, which are more electron rich than the B linkers in PT_2_B- and PY_2_B-COFs. Scale bars: **a**: 20 nm; **c**, **d**, **h**: 50 nm

TEM data (Fig. 6 and Supplementary Fig. 17) provide strong support for the structural model (see Supplementary Fig. 18 for SEM measurements). Different *d*-spacings can be observed in the same sample—and often in the same crystallite—strongly suggesting a structural model based on a unit cell with significantly different *a* and *b* parameters describing the in-plane structure. The observed crystallites, in many cases bent, feature a large repeat unit of 3.9–4.1 nm for PT_2_B- and PY_2_B-COFs (Fig. 6d, f) corresponding to the edge distance *a*, which at 4.8–5.0 nm is longer for PT- and PY-COFs (Fig. 6h, i). The other prominently observed *d*-spacing is much smaller at ~1.9 nm (Fig. 6e) and is similar for all four COFs and corresponds to the unit cell length *b*. The shorter edge lengths in TEM measurements, as compared with those observed with PXRD measurements, is known to result from shrinkage of the structure owing to damage by the electron beam^42^. In addition, in the [100] direction, while PT_2_B- and PY_2_B-COFs show a monomodal intensity variation (Fig. 6d, f, g), PT- and PY-COFs exhibit a bimodal intensity variation (Fig. 6h–j) in each unit cell as a result of the higher electron density corresponding to the pyrene moieties between the ribbons in the latter systems, as also verified by simulated potential maps of the model structures. The COF crystallites also possess a large anisotropy and differ significantly in their extension in [100] and [010] directions (Supplementary Table 4). Elongated strip-like crystallites can be observed with predominant extension along [010] direction, i.e., the direction of *b*. This demonstrates that the ribbons can act as supramolecular motifs, i.e., secondary building units for hierarchical design of differently expanded frameworks. Along [100] or the direction of *a*, the crystallites are much shorter. This is especially true for PT- and PY-COFs. It should be noted that such detailed TEM analysis of COFs is rare^42,51,52^ as useful measurements are typically impeded by the low TEM contrast and most importantly, because of an easy degradation of most COFs under the electron beam, for most except highly crystalline samples^42^.

### Surface area and porosity measurements

The higher crystallinity of PT_2_B- and PY_2_B-COFs is also reflected in their higher Brunauer–Emmett–Teller (BET) surface area as evaluated by measuring Argon sorption isotherms at 87 K (Figs. 3d, 5d). Although the BET surface areas of the PT- and PY-COFs are 1791 m^2^ g^−1^ and 1220 m^2^ g ^−1^, respectively, those for the PT_2_B- and PY_2_B-COFs are 2367 m^2^ g^−1^ and 1984 m^2^ g^−1^, respectively. The BET surface areas of PT_2_B- and PY_2_B-COFs are comparable to some of the most porous 2D COFs, such as ILCOF-1 (2723 m^2^ g^−1^)^53^, 4PE-1P-COF (2140 m^2^ g^−1^)^54^ and TPB-DMTP-COF (2015 m^2^ g^−1^)^55^. The absence of free amine functional groups in PT_2_B- and PY_2_B-COFs also reduces the possibility of chemisorbed molecules clogging the pores and possibly contributes to the larger surface area. A pore size distribution (PSD) was calculated from the Argon sorption isotherms using a Quenched Solid DFT adsorption kernel for Argon at 87 K on carbon with cylindrical pores. For the PT- and PY-COFs, only one type of pore centered at 1.77–1.83 nm is observed (Supplementary Fig. 19a). This matches with the theoretical pore sizes predicted from the crystal structures (Supplementary Fig. 20), with the  larger  pore having pendant amine functionalities behaving as two smaller pores having sizes closer to the small pores in the ribbons. On the other hand, owing to the absence of the pendant amine functionalities and hence a larger second pore, two distinct pores can be seen in the PSD for PT_2_B- and PY_2_B-COFs at ~1.83 nm and 2.37 nm (Supplementary Fig. 19b), again in agreement with crystal structure predictions. The pore volumes for PT- and PY-COFs are 0.896 cm^3^ g^−1^ and 0.837 cm^3^ g^−1^, respectively. The pore volumes for PT_2_B- and PY_2_B-COFs are larger at 1.03 cm^3^ g^−1^ and 1.259 cm^3^ g^−1^, respectively.

### Photoluminescence measurements

The COFs being distinctly emissive when dispersed in acetonitrile (Supplementary Fig. 21 and Supplementary Table 5), the presence of free amine functionalities can be corroborated with photoluminescence measurements. The detailed photophysical characterizations are listed in Supplementary Table 5. For PT- and PY-COFs in acetonitrile with ~2 equivalents of 2,6-lutidine (per –NH_2_ group) as the base for trapping the HI generated, the emission intensity first drops and then increases beyond the initial value on addition of increasing amounts of methyl iodide (MeI) (Supplementary Fig. 22, 23). The drop in emission intensity can be related to the initial formation of –NHMe and -NMe_2_ species which further increase the photoinduced electron transfer quenching of pyrene-based fluorescence by the donor amine^56^. As the concentration of MeI is increased further, -NMe_3_^+^ species form, which cannot participate in electron transfer quenching anymore and thus the emission intensity increases. On the other hand, no such obvious trend was observed for PT_2_B-COF, which does not have the free amine functional moieties in it. PY_2_B-COF was too weakly emissive to be studied. The change in the photoluminescence emission intensity thus substantiates the presence of a COF structure with free amine groups.

### CO_2_ adsorption studies

Free amine functional groups are known to enhance CO_2_ adsorption capacity of porous frameworks and could thus again corroborate their presence^57–60^. Thus, CO_2_ sorption isotherms were recorded at 273 K and the absolute CO_2_ uptake capacities of PT-, PY-, PT_2_B-, and PY_2_B-COFs at 1 atm are found to be 95, 146, 85, and 127 mg g^−1^, respectively. To put this in perspective, the CO_2_ uptake capacity of highly active carboxylic acid functionalized porphyrin based H_2_P-COFs reported by Jiang and co-workers is in the range 96–174 mg g^−1^ ^26^. CO_2_ uptake capacity normalized to the BET surface area of the individual samples shows a clear trend (Supplementary Fig. 24): PT- and PY-COFs have 1.46 and 1.67 times higher CO_2_ adsorption capacity than PT_2_B- and PY_2_B-COFs, respectively, an effect attributed predominantly to the presence of free amine groups.

### Heterogeneous organocatalysis

The presence of these free amine functionalities in the COF framework could be further substantiated by employing them as heterogeneous organocatalysts. The aromatic amine-catalyzed cyclization–substitution cascade reaction of 2-hydroxylcinnamaldehyde with trimethylsilyl enol ether to form substituted chromenes was chosen because no radical intermediates are known to be involved and the reaction takes place at room temperature^61^. For PT_2_B- and PY_2_B-COFs, product formation is negligible (1–2% only). On the other hand, with PT- and PY-COFs, the reaction yields of 22% and 8%, respectively, with excellent regioselectivity (Supplementary Table 6, Supplementary Fig. 25–28), show that these COFs are porous heterogeneous amine-group terminated materials indeed. It is important to consider the proposed mechanism for the aforesaid catalytic reaction according to which a Schiff base adduct is initially formed in situ between the pendant amine groups and 2-hydroxylcinnamaldehyde (Supplementary Fig. 29)^61^. The fact that the reaction works with COFs as the amine catalysts thus notably shows that the pendant amine moieties in the pores can actually be modified suitably and reversibly in the COF template and can be used to link functional moieties specific to particular applications.

### Functional group interconversion

**Fig. 7:**
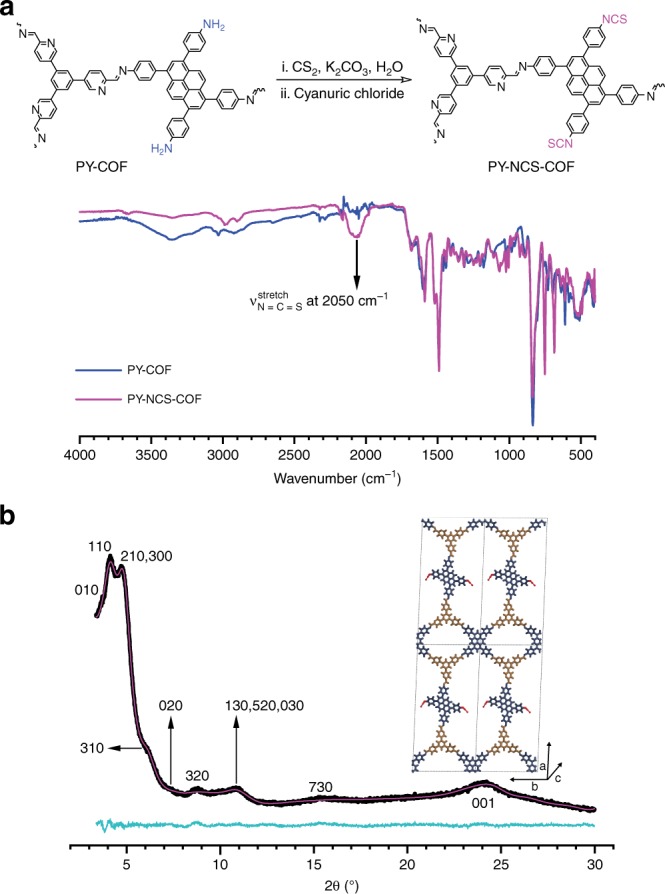
Post-crystallization amine to isothiocyanate group interconversion reaction with PY-COF. **a** FT-IR spectrum of PY-NCS- and PY-COF showing the appearance of ν_N=C =S(stretch)_ signal post modification. **b** PXRD pattern of PY-NCS-COF with corresponding Rietveld refinement (magenta) showing good fits to the experimental data (black) with minimal differences. The turquoise trace shows the difference between the experimental data and the refinement. The corresponding Miller indices of the identifiable reflections are shown as well. The inset shows an illustration of the COF structure model (viewed along the stacking direction)

**Fig. 8:**
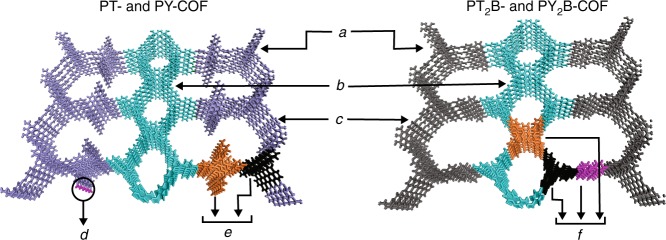
Summary of the unique features of the sub-stoichiometric and the three-component COFs. **a** *bex* network topology. **b** Supramolecular ribbon-like motifs as secondary building units for hierarchical design. **c** Highly crystalline and porous, dual pore COFs. **d** Free functional groups by sub-stoichiometric design—free amines in an imine-linked COF, functionalizable, and catalytically active. **e** Combination of tetratopic and triangular linkers—dual linking mode of tetratopic molecule. **f** Combination of di-, tri-, and tetratopic linkers

As a proof-of-concept, we explored a functional group interconversion reaction, namely the conversion of amines to isothiocyanates. The COF as the amine substrate was reacted with CS_2_ to generate the corresponding dithiocarbamate salt in situ followed by elimination to the isothiocyanate product using cyanuric chloride as the desulfurylation reagent (Figs 7, 8, and Supplementary Methods)^62,63^. Fig. 7a compares the FT-IR spectrum of PY-COF and PY-NCS**-**COF. The *ν*_N-H(stretch)_ in the region 3430 cm^−1^–3190 cm^−1^ corresponding to the free amines in PY-COF is found to be almost absent in PY-NCS-COF. This is accompanied with the appearance of *ν*_N=C=S(stretch)_ signal at 2050 cm^−1^. ^15^N NMR spectrum (Supplementary Fig. 30) again shows a diminished intensity of the amine signal at − 330 ppm together with the concurrent appearance of a weak, but prominent signal at −274 ppm corresponding to the nitrogen atom of the isothiocyanate group. The chemical shift of this signal matches well with the ^15^N NMR signal for PhNCS. From the ^13^C spectrum (Supplementary Fig. 31) it is difficult to identify a signal due to the isothiocyanate carbon (see Supplementary Fig. 32 for EDX analysis); it is also known for the isothiocyanate carbon to be near-silent or not observed at all in ^13^C NMR spectra^64–67^. Interestingly, although there is a slight broadening of reflections, the PXRD pattern of PY-NCS-COF shows that crystallinity is retained in this functional group transformation reaction (Fig. 5b). Similar to PY-COF, the experimental PXRD pattern shows eight peaks at 3.7, 4.1, 4.7, 6.1, 7.3, 8.7, 10.9, and 15.2° 2*θ*, together with the broad stacking reflection at 2*θ* = 24°. The changes in reflection intensities as compared to PY-COF could be successfully modeled by replacing the dangling amine groups with isothiocyanate functional groups during Rietveld refinement, further validating the structural transformation. Rietveld refinement of the experimental powder pattern resulted in unit cell parameters of *a* = 53.024 Å, *b* = 23.551 Å, *c* = 3.794 Å, and *α* = *β* = 90°, *γ* = 92.106°, *R*_wp_ = 1.62 (Supplementary Data 5). From the Argon sorption isotherm at 87 K (Supplementary Fig. 33), the BET surface area of PY-NCS-COF is measured to be 979 m^2^ g^−1^. Thus, a complete topochemical functional group interconversion could be achieved with our sub-stoichiometric COFs.

## Discussion

We report sub-stoichiometric COFs (Figure 8), by demonstrating an unconventional combination of tritopic and tetratopic linkers to form a hitherto unexpected 2D COF of a previously unidentified network topology where the pyrene-based linker P uniquely assumes a simultaneous dual role of di- and tetratopic linker in two different coordination environments. The presence of periodic uncondensed amine functional groups in the imine-linked sub-stoichiometric PT- and PY-COFs is a principal observation with immediate consequences for both the design principles and functionalization of COFs: this design concept goes beyond conventional post-synthetic functionalization, while still retaining its essence.

Although we have demonstrated this concept for imine-linked COFs, it may be extended to other COF linkages as well. The implication of these results is profound; it shows that complex structures can be achieved by unusual linker combinations, without having to pursue arduous synthesis of sophisticated linkers. For example, in an analogous way, the combination of appropriate hexatopic and tetratopic linkers can be envisaged to produce a sub-stoichiometric COF (Supplementary Fig. 34). Our result shows that by just considering the linker geometry, it may not always be possible to predict the correct COF structure and it prompts us to think beyond conventional retrosynthetic topological deconstruction rules for designing COFs and to create a new class of structures with unique complexities, including possibly quasicrystalline COFs (Supplementary Fig. 35). Most importantly, such sub-stoichiometric COFs are characteristically linked to free functional groups (without the need of separate functionalization of the linkers), which can either be designed for use as such, or can be further derivatized for specific applications with the COF as the template, which again opens up a new direction of research. For example, the free amine moieties can be used to link molecular proton reduction co-catalysts to increase the photocatalytic H_2_ evolution efficiency^68^. The possibilities are numerous.

## Methods

### Synthesis of PT-/PY-COF

P (0.076 mmol, 43.2 mg), T/Y (0.076 mmol, 30 mg), 1,4-dioxane (3 ml), mesitylene (3 ml), and aqueous acetic acid (6 M, 0.6 ml) were added to a Biotage© precision glass vial, sealed, and heated under autogenous pressure at 120 °C for 72 h. After the reaction was allowed to cool down, the reaction mixture was filtered and washed thoroughly with water, tetrahydrofuran, chloroform, and acetone, and then dried in high dynamic vacuum overnight to get PT- and PY-COF in 72% and 68% yield, respectively.

### Synthesis of PT_2_B-/PY_2_B-COF

P (0.064 mmol, 36 mg), T/Y (0.127 mmol, 50 mg), B (0.064 mmol, 12 mg), 1,4-dioxane (5 ml), mesitylene (5 ml), and aqueous acetic acid (6 M, 1 ml) were added to a Biotage© precision glass vial, sealed, and heated under autogenous pressure at 120 °C for 72 h. After the reaction was allowed to cool down, the reaction mixture was filtered and washed thoroughly with water, tetrahydrofuran, chloroform, and acetone and then dried in high dynamic vacuum overnight overnight to get PT_2_B- and PY_2_B-COF in 75% and 72% yield, respectively.

### Synthesis of PY-NCS-COF

To a solution of K_2_CO_3_ (20 mg, 0.145 mmol) in water (10 ml), 50 mg of PY-COF was added. To the resulting suspension, CS_2_ (40 µl, 0.66 mmol) was added dropwise. After the addition was complete, the reaction mixture was stirred overnight at room temperature. The reaction mixture was then cooled to 0 ^o^C and a solution of cyanuric chloride (5.1 mg, 0.0276 mmol) in 5 ml dichloromethane was added dropwise. After complete addition, the reaction mixture was stirred for 3 h and then filtered. The residue was basified with 6 N NaOH solution and then washed with water until the washings were pH neutral followed by washing with acetone, chloroform, and tetrahydrofuran and then dried in high dynamic vacuum overnight to get PY-NCS-COF in 62% yield.

## Supplementary information

Supplementary Information

Description of Additional Supplementary Files

Supplementary Data 1

Supplementary Data 2

Supplementary Data 3

Supplementary Data 4

Supplementary Data 5

## Data Availability

All data supporting the findings of this study are available within the Article and its Supplementary Information and/or from the corresponding authors upon reasonable request. Reprints and permissions information is available online at www.nature.com/reprints.

## References

[1] Cote AP (2005). Porous, crystalline, covalent organic frameworks. Science.

[2] Huang N, Wang P, Jiang D (2016). Covalent organic frameworks: a materials platform for structural and functional designs. Nat. Rev. Mater..

[3] Diercks CS, Yaghi OM (2017). The atom, the molecule, and the covalent organic framework. Science.

[4] Bisbey RP, Dichtel WR (2017). Covalent organic frameworks as a platform for multidimensional polymerization. ACS Cent. Sci..

[5] Lohse, M. S. & Bein, T. Covalent organic frameworks: structures, synthesis, and applications. *Adv. Funct. Mater*. **28**, 1705553 (2018).

[6] El.-Kaderi HM (2007). Designed synthesis of 3D covalent organic frameworks. Science.

[7] Jin Y, Hu Y, Zhang W (2017). Tessellated multiporous two-dimensional covalent organic frameworks. Nat. Rev. Chem..

[8] Waller PJ, Gandara F, Yaghi OM (2015). Chemistry of covalent organic frameworks. Acc. Chem. Res..

[9] Yaghi OM (2003). Reticular synthesis and the design of new materials. Nature.

[10] Qian C (2018). A design strategy for the construction of 2D heteropore covalent organic frameworks based on the combination of *C*_*2v*_ and *D*_*3h*_ symmetric building blocks. Polym. Chem..

[11] Xu S-Q, Zhan T-G, Wen Q, Pang Z-F, Zhao X (2016). Diversity of covalent organic frameworks (COFs): a 2D COF containing two kinds of triangular micropores of different sizes. ACS Macro Lett..

[12] Baldwin LA, Crowe JW, Shannon MD, Jaroniec CP, McGrier PL (2015). 2D covalent organic frameworks with alternating triangular and hexagonal pores. Chem. Mater..

[13] Crowe JW, Baldwin LA, McGrier PL (2016). Luminescent covalent organic frameworks containing a homogeneous and heterogeneous distribution of dehydrobenzoannulene vertex units. J. Am. Chem. Soc..

[14] Yang H (2015). Mesoporous 2D covalent organic frameworks based on shape-persistent arylene-ethynylene macrocycles. Chem. Sci..

[15] Cai S–L (2016). Rationally designed 2D covalent organic framework with a brick-wall topology. ACS Macro Lett..

[16] Qian C, Xu S-Q, Jiang G-F, Zhan T-G, Zhao X (2016). Precision construction of 2D heteropore covalent organic frameworks by a multiple-linking-site strategy. Chem. Eur. J..

[17] Tian Y (2016). Two-dimensional dual-pore covalent organic frameworks obtained from the combination of two *D*_*2h*_ symmetrical building blocks. Chem. Commun..

[18] Qian C (2017). Toward covalent organic frameworks bearing three different kinds of pores: the strategy for construction and COF-to-COF transformation *via* heterogeneous linker exchange. J. Am. Chem. Soc..

[19] Yin Z-J (2017). Ultrahigh volatile iodine uptake by hollow microspheres formed from a heteropore covalent organic framework. Chem. Commun..

[20] Tian Y (2017). Construction of two heteropore covalent organic frameworks with Kagome lattices. CrystEngComm.

[21] Huang NL (2016). Multiple-component covalent organic frameworks. Nat. Commun..

[22] Pang Z–F (2016). Construction of covalent organic frameworks bearing three different kinds of pores through the heterostructural mixed linker strategy. J. Am. Chem. Soc..

[23] Dalapati S, Jin E, Addicoat M, Heine T, Jiang D (2016). Highly emissive covalent organic frameworks. J. Am. Chem. Soc..

[24] Ding S–Y (2016). Thioether-based fluorescent covalent organic framework for selective detection and facile removal of mercury(II). J. Am. Chem. Soc..

[25] Doonan CJ, Tranchemontagne DJ, Glover TG, Hunt JR, Yaghi OM (2010). Exceptional ammonia uptake by a covalent organic framework. Nat. Chem..

[26] Huang N, Chen X, Krishna R, Jiang D (2015). Two-dimensional covalent organic frameworks for carbon dioxide capture through channel-wall functionalization. Angew. Chem. Int. Ed..

[27] Vyas VS (2015). A tunable azine covalent organic framework platform for visible light-induced hydrogen generation. Nat. Commun..

[28] Banerjee T, Gottschling K, Savasci G, Ochsenfeld C, Lotsch B (2018). H_2_ evolution with covalent organic framework photocatalysts. ACS Energy Lett..

[29] Wei P-F (2018). Benzoxazole-linked ultrastable covalent organic frameworks for photocatalysis. J. Am. Chem. Soc..

[30] Xu H, Tao S, Jiang D (2016). Proton conduction in crystalline and porous covalent organic frameworks. Nat. Mater..

[31] Chandra S (2014). Phosphoric acid loaded azo (−N═N−) based covalent organic framework for proton conduction. J. Am. Chem. Soc..

[32] Dalapati S (2015). Rational design of crystalline supermicroporous covalent organic frameworks with triangular topologies. Nat. Commun..

[33] Mulzer CR (2016). Superior charge storage and power density of a conducting polymer-modified covalent organic framework. ACS Cent. Sci..

[34] Nagai A (2011). Pore surface engineering in covalent organic frameworks. Nat. Commun..

[35] Lohse MS (2016). Sequential pore wall modification in a covalent organic framework for application in lactic acid adsorption. Chem. Mater..

[36] Bunck DN, Dichtel WR (2012). Internal functionalization of three-dimensional covalent organic frameworks. Angew. Chem. Int. Ed..

[37] Brucks SD, Bunck DN, Dichtel WR (2014). Functionalization of 3D covalent organic frameworks using monofunctional boronic acids. Polymer.

[38] Gao Q (2018). Covalent organic framework with frustrated bonding network for enhanced carbon dioxide storage. Chem. Mater..

[39] Zeng Y (2015). Covalent organic frameworks formed with two types of covalent bonds based on orthogonal reactions. J. Am. Chem. Soc..

[40] Haase F, Banerjee T, Savasci G, Ochsenfeld C, Lotsch BV (2017). Structure−property−activity relationships in a pyridine containing azine-linked covalent organic framework for photocatalytic hydrogen evolution. Faraday Discuss..

[41] Vyas VS (2016). Exploiting noncovalent interactions in an imine-based covalent organic framework for quercetin delivery. Adv. Mater..

[42] Haase F (2018). Topochemical conversion of an imine- into a thiazole-linked covalent organic framework enabling real structure analysis. Nat. Commun..

[43] Wang Y, Liu H, Zhang X, Zhang Z, Huang D (2017). Experimental and mechanistic insights into copper(II)–dioxygen catalyzed oxidative N-dealkylation of N-(2-yridylmethyl)phenylamine and its derivatives. Org. Biomol. Chem..

[44] Xie H, Liao Y, Chen S, Chen Y, Deng G-J (2015). Copper-catalyzed efficient direct amidation of 2-methylquinolines with amines. Org. Biomol. Chem..

[45] Padhi SK, Manivannan V (2006). Cu(NO_3_)_2_‚3H_2_O-mediated synthesis of 4′-(2-Pyridyl)-2,2′:6′,2′′-terpyridine (L2) from N-(2-Pyridylmethyl)pyridine-2-methylketimine (L1). A C−C bond-forming reaction and the structure of{[Cu(L2)(OH)(NO_3_)][Cu(L2)(NO_3_)_2_]}.2H_2_O. Inorg. Chem..

[46] Liang J, Lv J, Shang Z-C (2011). Metal-free synthesis of amides by oxidative amidation of aldehydes with amines in PEG/oxidant system. Tetrahedron.

[47] Miyamura H, Min H, Soule J-F, Kobayashi S (2015). Size of gold nanoparticles driving selective amide synthesis through aerobic condensation of aldehydes and amines. Angew. Chem. Int. Ed..

[48] O’Keeffe M, Peskov MA, Ramsden SJ, Yaghi OM (2008). The reticular chemistry structure resource (RCSR) database of, and symbols for, crystal nets. Acc. Chem. Res..

[49] Chui SS-Y, Lo SM-F, Charmant JPH, Orpen AG, Williams ID (1999). A Chemically functionalizable nanoporous material [Cu_3_(TMA)_2_(H_2_O)_3_]_n_. Science.

[50] Lan Y (2018). Materials genomics methods for high-throughput construction of COFs and targeted synthesis. Nat. Commun..

[51] Haase F (2017). Tuning the stacking behaviour of a 2D covalent organic framework through non-covalent interactions. Mater. Chem. Front.

[52] Liu Y (2016). Weaving of organic threads into a crystalline covalent organic framework. Science.

[53] Rabbani MG (2013). A 2D mesoporous imine-linked covalent organic framework for high pressure gas storage applications. Chem. Eur. J..

[54] Ascherl L (2016). Molecular docking sites designed for the generation of highly crystalline covalent organic frameworks. Nat. Chem..

[55] Xu H, Gao J, Jiang D (2015). Stable, crystalline, porous, covalent organic frameworks as a platform for chiral organocatalysts. Nat. Chem..

[56] Lakowicz, J. R. *Principles of Fluorescence Spectroscopy*, Ch. 9 (Springer, New York, 2006).

[57] Huang N, Krishna R, Jiang D (2015). Tailor-made pore surface engineering in covalent organic frameworks: systematic functionalization for performance screening. J. Am. Chem. Soc..

[58] Vaidhyanathan R (2010). Direct observation and quantification of CO_2_ binding within an amine-functionalized nanoporous solid. Science.

[59] Couck S (2009). An amine-functionalized MIL-53 metal-organic framework with large separation power for CO_2_ and CH_4_. J. Am. Chem. Soc..

[60] Stavitski E (2011). Complexity behind CO_2_ Capture on NH_2_-MIL-53(Al). Langmuir.

[61] Yu C (2016). Aniline-promoted cyclization–replacement cascade reactions of 2-hydroxycinnamaldehydes with various carbonic nucleophiles through in situ formed N,O-acetals. Chem. Eur. J..

[62] Sun N (2012). A general and facile one-pot process of isothiocyanates from amines under aqueous conditions. Beilstein J. Org. Chem..

[63] Chen Y, Su L, Yang X, Pan W, Fang H (2015). Enantioselective synthesis of 3,5-disubstituted thiohydantoins and hydantoins. Tetrahedron.

[64] Spectral Database for Organic Compounds, SDBSWeb, National Institute of Advanced Industrial Science and Technology (AIST), http://sdbs.db.aist.go.jp. Propyl isothiocyanate: SDBS No. – 21756, Phenyl isothiocyanate: SDBS No. – 4809, *m*-tolyl isothiocyanate: SDBS No. – 19528, *p*-tolyl isothiocyanate: SDBS No. – 233.

[65] Glaser R (2015). Near-silence of isothiocyanate carbon in ^13^C NMR spectra: a case study of allyl isothiocyanate. J. Org. Chem..

[66] Jones RG, Allen G (1982). Carbon-13 NMR spectra of a series of *para*-substituted phenyl isothiocyanates. Org. Magn. Reson..

[67] Giffard M, Cousseau J, Martin GJ (1985). A comparative multinuclear ^l^H, ^13^C, and ^15^N magnetic resonance study of organic thiocyanates and lsothiocyanates. J. Chem. Soc. Perkin Trans..

[68] Elahipanah S, O’Brien PJ, Rogozhnikov D, Yousaf MN (2017). General dialdehyde click chemistry for amine bioconjugation. Bioconjugate Chem..

