# Publisher Correction: Sub-stoichiometric 2D covalent organic frameworks from tri- and tetratopic linkers

**DOI:** 10.1038/s41467-019-11188-8

**Published:** 2019-07-10

**Authors:** Tanmay Banerjee, Frederik Haase, Stefan Trenker, Bishnu P. Biswal, Gökcen Savasci, Viola Duppel, Igor Moudrakovski, Christian Ochsenfeld, Bettina V. Lotsch

**Affiliations:** 1 Max Planck Institute for Solid State Research, Heisenbergstraße 1, 70569 Stuttgart, Germany; 2 Department of Chemistry, University of Munich (LMU), Butenandtstraße 5-13, 81377 München, Germany; 3 Cluster of Excellence e-conversion, Schellingstraße 4, 80799 München, Germany; 4 Center for Nanoscience, Schellingstraße 4, 80799 München, Germany; 5 Present Address: Institute for Integrated Cell-Material Sciences (WPI-iCeMS), Kyoto University, Kyoto, 606-8501 Japan

**Keywords:** Polymers, Porous materials, Polymers

Correction to: *Nature Communications* 10.1038/s41467-019-10574-6, published online 19 July 2019.

The original version of this Article contained an error in the legend of Fig. 5a, in which the blue data was incorrectly labelled ‘PY_2_B-COF’ rather than the correct ‘PT_2_B-COF’ and the grey data was incorrectly labelled ‘PT_2_B-COF’ rather than the correct ‘PY_2_B-COF’. This has been corrected in both the PDF and HTML versions of the Article.

