# Supplementary material for: Sub-stoichiometric 2D covalent organic frameworks from tri- and tetratopic linkers
Source: Nat Commun. 2019 Jun 19;10:2689. doi: 10.1038/s41467-019-10574-6 (PMC6584614; doi:10.1038/s41467-019-10574-6)
Supplement: Supplementary file 1 — Supplementary Information [file 41467_2019_10574_MOESM1_ESM.pdf]

Supplementary Information

Sub-Stoichiometric 2D Covalent Organic Frameworks from Tri- and Tetratopic Linkers

Banerjee *et al.*

Table of contents

		Page
**Supplementary Methods**	…..	4
**Supplementary Figure 1.** FT-IR spectra of the linkers P, T and Y	…..	8
**Supplementary Figure 2.** ^{15}N ssNMR spectra of PT- and PY-COFs with corresponding assignments	…..	8
**Supplementary Figure 3**. ^{15}N ssNMR of benzidine	…..	9
**Supplementary Figure 4-9, Supplementary Table 1,2.** Quantum-chemical calculations	…..	10
**Supplementary Figure 10.** ^{13}C ssNMR spectra of PY- and PY_2B-COFs	…..	13
**Supplementary Figure 11**. Chemical stability tests of the COFs	…..	13
**Supplementary Table 3.** Pawley and Rietveld refinement of the different COFs with the respective unit cell parameters	…..	14
**Supplementary Figure 12.** Simulated PXRD patterns for AB stacking in PT-COF	…..	15
**Supplementary Figure 13**. Possible formation of 3D **tbo** net from tri- and tetratopic linkers	…..	16
**Supplementary Figure 14.** Comparison of the experimental PXRD pattern of PY-COF with the simulated patterns for 3D **tbo** and 2D **bex** nets	…..	16
**Supplementary Figure 15**. ^{15}N ssNMR spectra of PT_2B- and PY_2B-COFs with corresponding assignments	…..	17
**Supplementary Figure 16**. Simulated PXRD patterns for AB stacking in PT_2B-COF	…..	18
**Supplementary Figure 17**. TEM images COF crystallites	…..	19
**Supplementary Figure 18.** SEM images of COFs	…..	20
**Supplementary Table 4**. Dimensions of the anisotropic COF crystallites as observed with TEM	…..	21
**Supplementary Figure 19.** Pore size distributions calculated from Ar sorption isotherms	…..	21
**Supplementary Figure 20**. Theoretical pore diameters as obtained from the crystal structure model of the COFs	…..	22

Supplementary Figure 21. Diffuse reflectance and photoluminescence spectra of COFs ….. 23

Supplementary Table 5. Photophysical properties of COFs ….. 23

Supplementary Figure 22. Photoinduced electron transfer quenching studies of the COFs ….. 24

Supplementary Figure 23. Post photoluminescence PXRD of COFs ….. 25

Supplementary Figure 24. CO_2 sorption isotherms of the COFs at 273 K normalized to BET surface area ….. 25

Supplementary Table 6. Reaction yield and regioselectivity for the heterogeneous organocatalysis reaction with COF ….. 26

Supplementary Figure 25. ^{1}H NMR spectra of the isolated chromene product C1 ….. 27

Supplementary Figure 26. ^{1}H NMR spectrum of the crude reaction product using PT-COF as the aromatic amine catalyst ….. 28

Supplementary Figure 27. ^{1}H NMR spectrum of the crude reaction product using PY-COF as the aromatic amine catalyst ….. 29

Supplementary Figure 28. Post reaction PXRD patterns for PT- and PY-COFs ….. 30

Supplementary Figure 29. Mechanism of the COF-catalyzed reaction between 2-hydroxycinnamaldehyde and trimethylsilyl enol ether ….. 31

Supplementary Figure 30. ^{15}N ssNMR spectrum of PY-NCS-COF ….. 32

Supplementary Figure 31. ^{13}C ssNMR spectrum of PY-NCS-COF ….. 32

Supplementary Figure 32. EDX spectroscopic analysis of PY- and PY-NCS-COF ….. 33

Supplementary Figure 33. Argon sorption isotherm of PY-NCS-COF at 87 K ….. 33

Supplementary Figure 34. Proposed sub-stoichiometric COF composed of appropriate tetra- and hexatopic linkers ….. 34

Supplementary Figure 35. Proposed sub-stoichiometric quasicrystalline COF ….. 35

Supplementary References ….. 36

Supplementary Methods

Materials

Y,[1] P[2] and T[3] were synthesized and characterized following reported procedures. The analytical data match with those reported. B, 5-bromo-2-pyridinecarboxaldehyde, $Pd(PPh_3)_4$, 2-hydroxycinnamaldehyde, trimethylsilyl enol ether, 4Å molecular sieves, CS_2, cyanuric chloride, CH_3I and 2,6-lutidine were obtained from either Sigma-Aldrich or Fluka. Solvents were obtained from Merck and Roth.

Elemental analysis for PT-/PY-COF. Calculated for $(C_{128}H_{78}N_{14})_n$ C, 84.84; N, 10.82; H, 4.34. Found: For PT-COF: C, 78.60; N, 10.41; H, 4.13. For PY-COF: C, 74.95; N, 9.42; H, 4.91.

Elemental analysis for PT_2B-/PY_2B-COF. Calculated for $(C_{100}H_{60}N_{12})_n$ C, 84.01; N, 11.76; H, 4.23. Found: For PT_2B-COF: C, 78.80; N, 11.22; H, 4.07. For PY_2B-COF: C, 72.20; N, 9.77; H, 4.72.

For the following reaction conditions, PT-/PY-COF products were less or similarly crystalline:

For PT-COF : a. P:T = 3:4 in 1:1 mesitylene:dioxane; b. P:T = 3:2 in 1:1 mesitylene:dioxane; c. P:T = 3:8 in 1:1 mesitylene:dioxane; d. P:T = 1:1 in 2:1 mesitylene:dioxane; e. P:T = 1:1 in 1:2 mesitylene:dioxane; f. P:T = 1:1 in 1:9 mesitylene:dioxane; g. P:T = 1:1 in 9:1 mesitylene:dioxane; h. P:T = 1:1 in 7:3 *o*-dichlorobenzene:*n*-butanol.

For PY-COF: a. P:Y = 3:4 in 1:1 mesitylene:dioxane.

Synthesis of 2-(2H-chromen-2-yl)-1-phenylethan-1-one (C1) with COF-catalyst. To a solution of 2-hydroxycinnamaldehyde (5 mg, 0.034 mmol) in anhydrous dichloromethane (1 ml), COF (20 mol%) was added and stirred under Argon atmosphere. Activated 4Å molecular sieves (30 mg) and trimethylsilyl enol ether (8 µl, 0.037 mmol) were then added and stirred at room temperature for 48 hours. The reaction mixture was then filtered and evaporated to complete dryness and ^1H NMR was recorded using CH_2Br_2 internal standard. For a reference reaction with benzidine as the aromatic amine catalyst, a 56% reaction yield was obtained with a 14:1 r.r. For this reaction the product was isolated and the ^1H NMR spectrum is shown in Supplementary Figure 25.

PXRD

PXRD pattern were collected at room temperature on a Stoe Stadi P diffractometer (Cu-Kα1, Ge(111)) in Debye-Scherrer geometry. The sample was measured inside a sealed glass capillary (0.7 mm). For improved particle statistics the sample was spun.

Model Building

The structural models of PY- and PT-COF were built using the following key pieces of data:

1. Formation of a fully condensed tbo net was not supported by TEM measurements and XRPD data.

2. A broad reflection at 24° 2θ suggesting a 2D structure.

3. Approximate lattice spacings for two unequal directions (a and b) that were obtained from TEM.

With these informations, possible structural models were considered based on the geometry of the used building blocks and the possible molecular connectivity that were based on IR and ssNMR data. Feasible candidates were constructed in BIOVA Materials Studio 2017 (17.1.0.48. Copyright © 2016 Dassault Systèmes) suite and the structure and unit cell parameters were relaxed using force fields (Forcite, universal force fields with Ewald electrostatic and van der Waals summations method). The unit cells of these models were then refined with the experimentally obtained XRPD pattern. Rietveld refinement[4] was performed in the range 2-30° 2θ using Bruker-AXS TOPAS V5 2014 with fixed atom coordinates, and the models were compared based on the same set of refined variables. The peak profile was fitted using the fundamental parameter[5] approach as implemented in TOPAS. The background was modelled with a 6th order Chebychev polynomial. The highly anisotropic crystallite shape observed with TEM leading to peak broadening required the modelling of crystallite size and strain, with asymmetry adopted phenomenological model for microstrain and/or spherical harmonics.[6] The thus obtained structural models were then checked for bond length and bond angle consistency in the structure. The sub-stoichiometric nature of the thereby obtained models were further validated by ssNMR, IR, luminescence, Argon sorption measurements, and the comparison of simulated projected potential maps to the obtained TEM images.

The models for PY_2B- and PT_2B-COFs were built by substituting the 2-connected pyrene unit in the PY- and PT-COF models by benzidine. These models were then treated as the PY- and PT-COF models described before.

Quantum-chemical calculations

NMR chemical shifts were obtained on B97-2/pcS-2//PBE0-D3/def2-TZVP level of theory[7-12] using the Turbomole[13,14] program package in version 7.0.2 for geometries and the FermiONs++[15,16] program package for the calculation of NMR chemical shifts.

IR

Infrared spectra were recorded in attenuated total reflection (ATR) geometry on a PerkinElmer UATR Two equipped with a diamond crystal. The spectra were background corrected.

NMR

All liquid state NMR measurements were performed on a JEOL ECZ 400S 400 MHz spectrometer (magnetic field 9.4 T). ^{1}H, ^{13}C and ^{15}N measurements were performed in 5 mm NMR tubes using deuterium field lock. An appropriate number of accumulations have been made to achieve sufficient signal-to-noise ratio.

ssNMR was recorded on a Bruker Avance III 400 MHz spectrometer (magnetic field 9.4 T). For ssNMR spectroscopy, the samples were packed in 4 mm ZrO_2 rotors, which were spun in a Bruker WVT BL4 double resonance MAS probe. Chemical shift was referenced relative to tetramethylsilane (^{13}C), and CH_3NO_2 (^{15}N). The spinning rate was 12-14 kHz for ^{13}C NMR and 8 kHz for ^{15}N measurements. A standard cross-polarization sequence with a 2 ms ramped contact pulse was used for ^{13}C and a total of 4096-8192 scans were routinely accumulated. ^{15}N solid state NMR spectra were obtained with ramped cross-polarization and contact pulses of 3-8 ms optimized for the best signal. Both ^{13}C and ^{15}N measurements were performed in conditions of high-power broadband proton decoupling (SPINAL 64) with the spectral conditions being optimized for the shortest relaxation delay by measuring ^{1}H T_1 relaxation time.

SEM

SEM images were obtained on a Zeiss Merlin or a VEGA TS 5130MM (TESCAN) instrument with SEM-EDX measured using a Si/Li detector (10 kV acceleration voltage, Oxford).

TEM

TEM was performed with a Philips CM30 ST (300kV, LaB6 cathode). The samples were prepared dry onto a copper lacey carbon grid (Plano). The line scan analysis was done with ImageJ, version 1.52a. The simulation of the projected potential map was performed with the jems package (Stadelmann).

UV-Vis

Diffuse reflectance UV–visible absorption spectra were collected on a Cary 5000 spectrometer (referenced to barium sulphate). Absorption spectra were calculated from the reflectance data using the Kubelka-Munk function.

Steady-State and Time-Resolved Emission

Steady-state and time-resolved emission data were collected at room temperature using an Edinburgh FLS980 spectrometer. For steady-state emission, samples were excited using light output from a housed 450 W Xe lamp passed through a single grating (1800 l/mm, 250 nm blaze) Czerny-Turner monochromator and finally a bandwidth slit. Emission from the sample was passed through a double grating (1200 l/mm, 500 nm blaze) Czerny-Turner monochromator (appropriate bandwidth) and finally detected by a peltier-cooled Hamamatsu R928P photomultiplier tube.

The dynamics of emission decay were monitored by using the FLS980's time-correlated single-photon counting capability (1024 channels; 50 ns window) with data collection for 5000 counts. Excitation was provided by an Edinburgh EPL-375 picosecond pulsed laser diode (375 ± 6 nm, pulse width - 68 ps) and a cooled microchannel plate photomultipler tube (MCP-PMT) was used

as the detector. Kinetics were fit with a single exponential function by using Edinburgh software package, when required.

Emission quantum yields were acquired using an integrating sphere incorporated into a spectrofluorometer (FLS980, Edinburgh Instruments). The sample dispersions were placed in the sphere and a movable mirror was used for direct or indirect excitation, making it possible to measure absolute emission quantum efficiency following the De Mello method.[17] No bandpass filters were used during quantum yield measurements.

Sorption

Sorption measurements were performed on a Quantachrome Instruments Autosorb iQ MP with Argon at 87 K, and with CO_2 at 273 K. Samples were preheated in high vacuum at 120 °C for 12 h. BET surface areas were calculated from multiple data points from pressure ranges in accordance with IUPAC recommendations.[18] Pore size distributions were determined using the QSDFT kernel for Ar at 87 K on carbons (cylindrical pores, adsorption branch) implemented in the ASiQwin software v 3.01.

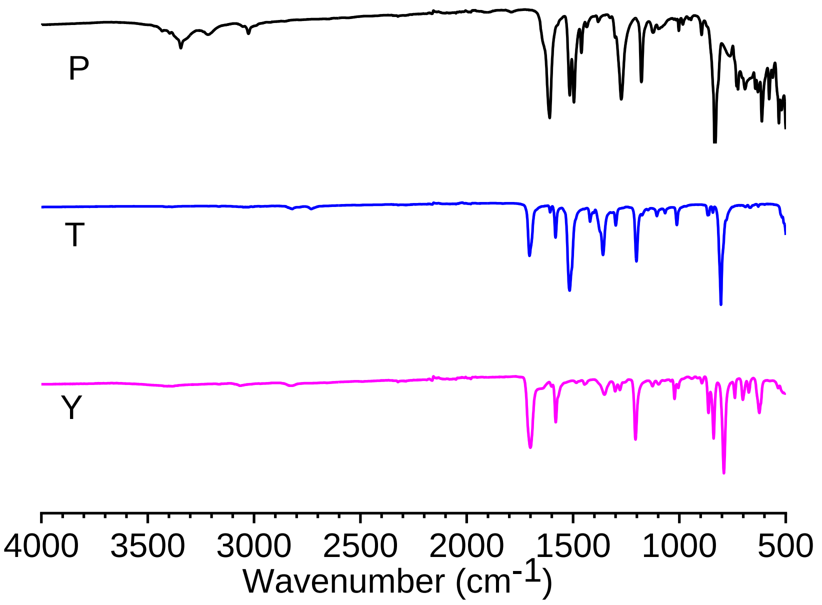

Supplementary Figure 1. FT-IR spectra of the linkers P, T and Y.

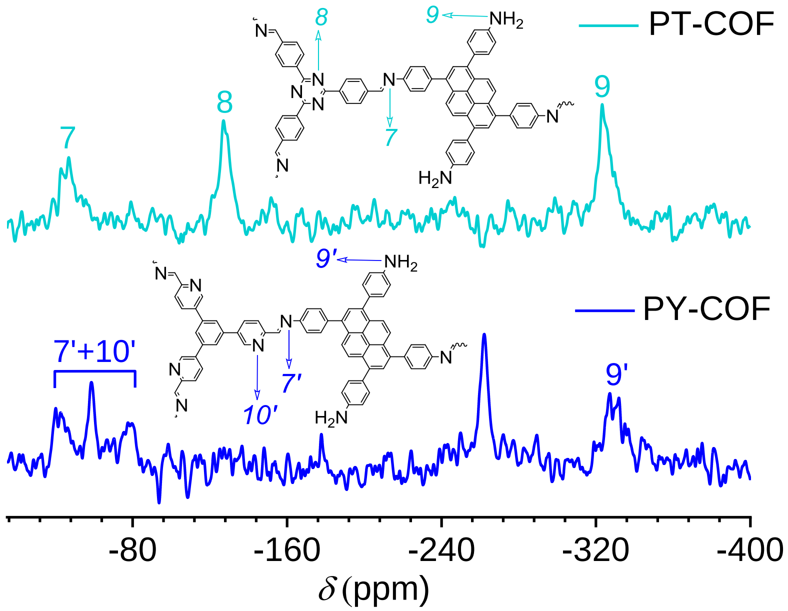

Supplementary Figure 2. ^{15}N ssNMR spectra of PT- and PY-COFs with corresponding assignments.

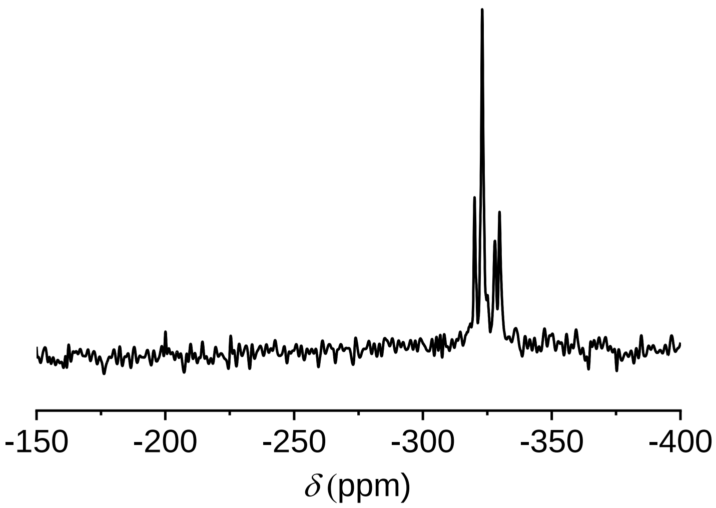

Supplementary Figure 3. ^{15}N ssNMR spectrum of benzidine.

Quantum-chemical calculations

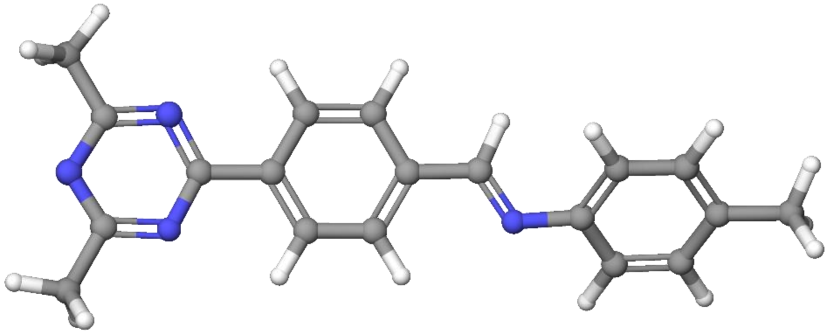

Supplementary Figure 4. Optimized geometry of the PT-COF and PT_2B-COF model system, obtained on PBE0-D3/def2-TZVP level of theory.

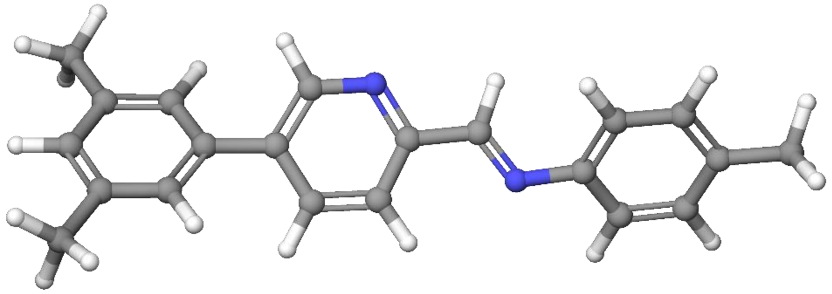

Supplementary Figure 5. Optimized geometry of the PY-COF-trans and PY_2B-COF-trans model systems with averted nitrogen atoms, obtained on PBE0-D3/def2-TZVP level of theory.

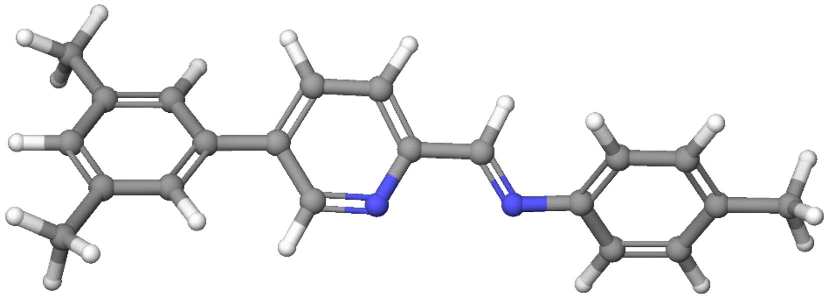

Supplementary Figure 6. Optimized geometry of the PY-COF-cis and PY_2B-COF-cis model system with facing nitrogen atoms, obtained on PBE0-D3/def2-TZVP level of theory.

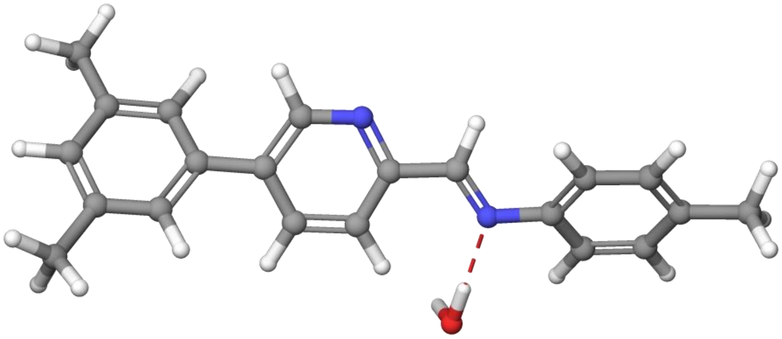

Supplementary Figure 7. Optimized geometry of the PY-COF-trans and PY_2B-COF-trans model system with the imine nitrogen atom H-bonded to H_2O, obtained on PBE0-D3/def2-TZVP level of theory.

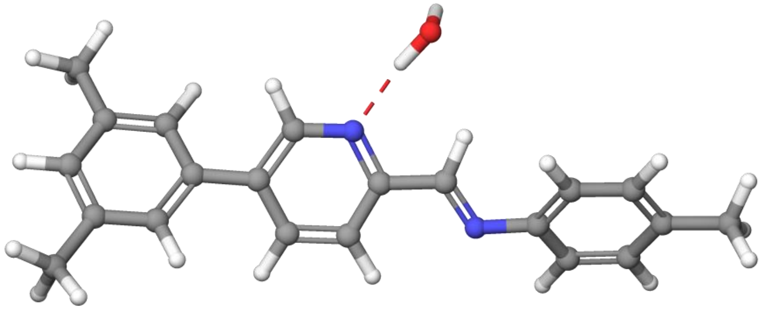

Supplementary Figure 8. Optimized geometry of the PY-COF-trans and PY_2B-COF-trans model system with the pyridine nitrogen atom H-bonded to H_2O, obtained on PBE0-D3/def2-TZVP level of theory.

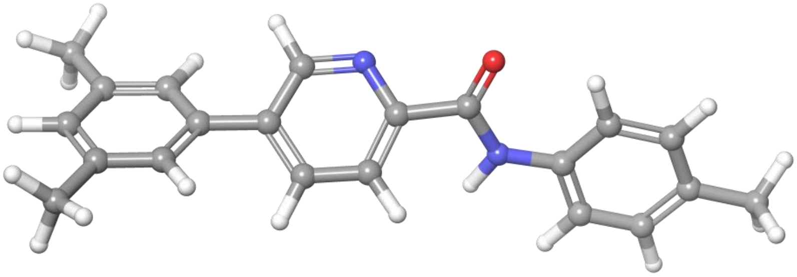

Supplementary Figure 9. Optimized geometry of the PY-COF and PY_2B-COF model system with amide linkage, obtained on PBE0-D3/def2-TZVP level of theory.

Supplementary Table 1. ^{15}N NMR chemical shifts for model systems, obtained on B97-2/pcS-2//PBE0-D3/def2-TZVP level of theory.

	^{15}N-NMR Chemical Shift [ppm]			
**Model**	**Imine nitrogen**	**Pyridine nitrogen**	**Triazine nitrogen**	**Amide nitrogen**
PT-COF, PT_2B-COF	-35.04	-	-121.37	-
PY-COF-trans, PY_2B-COF-trans	-36.12	-47.92	-	-
PY-COF-cis, PY_2B-COF-cis	-17.70	-48.02	-	-
imine H-bonded PY-COF-trans, PY_2B-COF-trans	-60.34	-46.71	-	-
pyridine H-bonded PY-COF-trans, PY_2B-COF-trans	-29.64	-72.16	-	-
PY-COF, PY_2B-COF with amide linkage	-	-45.38	-	-250.01

For PY- and PY_2B-COF, only the model systems with averted (trans) positions of the imine and pyridine nitrogen atoms (PY-COF-trans, PY_2B-COF-trans) are observed to fit the experimental ^{15}N NMR data.

Supplementary Table 2. ^{15}N NMR Chemical Shifts for selected small model systems, obtained on B97-2/pcS-2//PBE0-D3/def2-TZVP level of theory.

**Model**	**^{15}N-NMR Chemical Shift [ppm]**
Pyridine	-49.90
Protonated Pyridine	-198.64
Aniline	-332.34
Phenyl isothiocyanate	-280.83

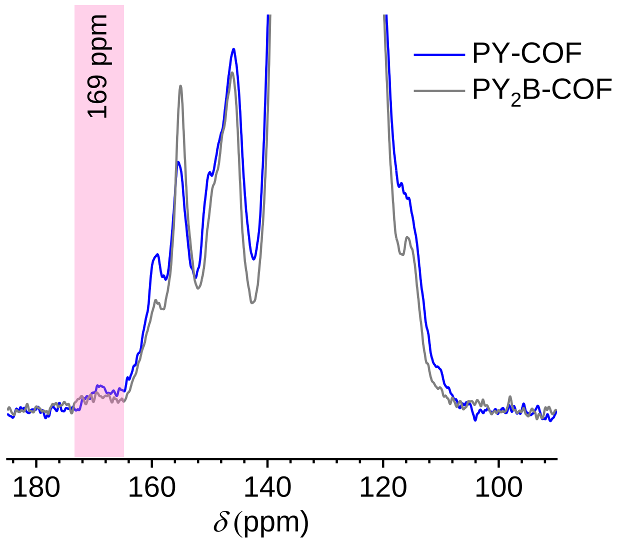

Supplementary Figure 10. ^{13}C ssNMR spectrum of PY- and PY_2B-COFs showing a possible amide carbonyl resonance at 169 ppm.

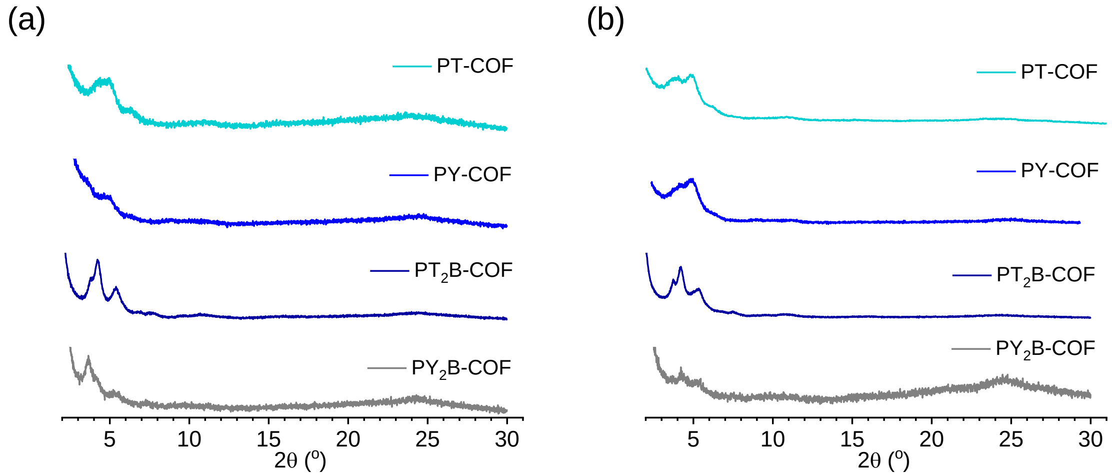

Supplementary Figure 11. Chemical stability tests of the COFs in (a) 1M aqueous HCl at room temperature for 30 hours, and (b) 1 N aqueous NaOH at room temperature for 30 hours. The COFs are reasonably stable in aqueous NaOH, except for PY_2B-COF. Acid treatment on the other hand leads to significant loss of crystallinity, except for PT_2B-COF. The instability of imine-bonded COFs in acidic and alkaline conditions has been previously reported.[19-21]

Supplementary Table 3. Pawley and Rietveld refinement of the different COFs with the respective unit cell parameters.

**COF**	**Refinement type**	R_{wp}	***a* (Å)**	***b*(Å)**	***c*(Å)**	α (°)	β (°)	γ (°)
PY	Pawley	1.307	52.710 ±0.445	24.239 ±0.160	3.8	90	90	93.108 ±0.481
	Rietveld	2.015	52.718 ±0.134	24.117 ±0.025	3.815 ±0.06	90	90	88.393 ±0.025
PT	Pawley	1.97	52.829 ±0.382	23.611 ±0.052	3.8	90	90	93.104 ±0.353
	Rietveld	3.222	52.516 ±0.073	23.556 ±0.029	3.681 ±0.182	90	90	90.759 ±0.056
PY_2B	Pawley	1.356	45.622 ±0.044	24.341 ±0.011	3.8	90	90	89.317 ±0.074
	Rietveld	2.587	45.845 ±0.052	24.352 ±0.017	3.743 ±0.025	90	90	90.981 ±0.135
PT_2B	Pawley	1.768	45.729 ±0.031	23.781 ±0.00004	3.8	90	90	92.111 ±0.183
	Rietveld	2.640	46.264 ±0.044	23.872 ±0.024	3.802 ±0.024	90	90	90.247 ±0.067

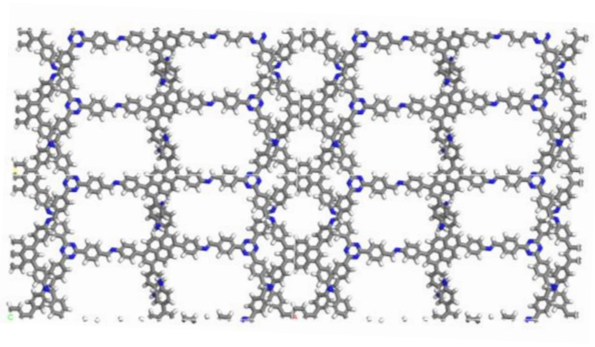

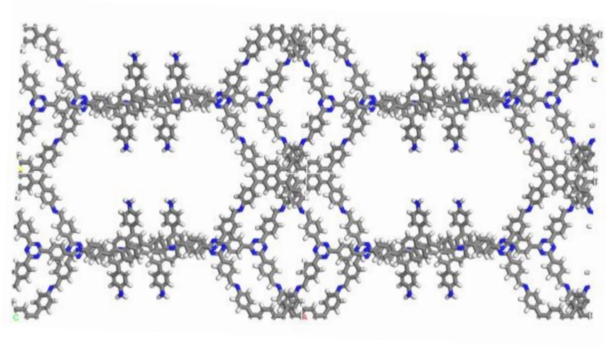

AB stacking, shifted along *b*

AB stacking, shifted along *a*

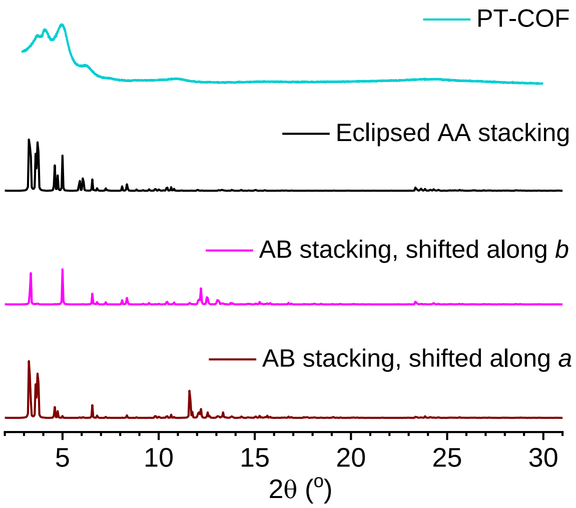

Supplementary Figure 12. Simulated PXRD patterns for AB stacking in PT-COF.

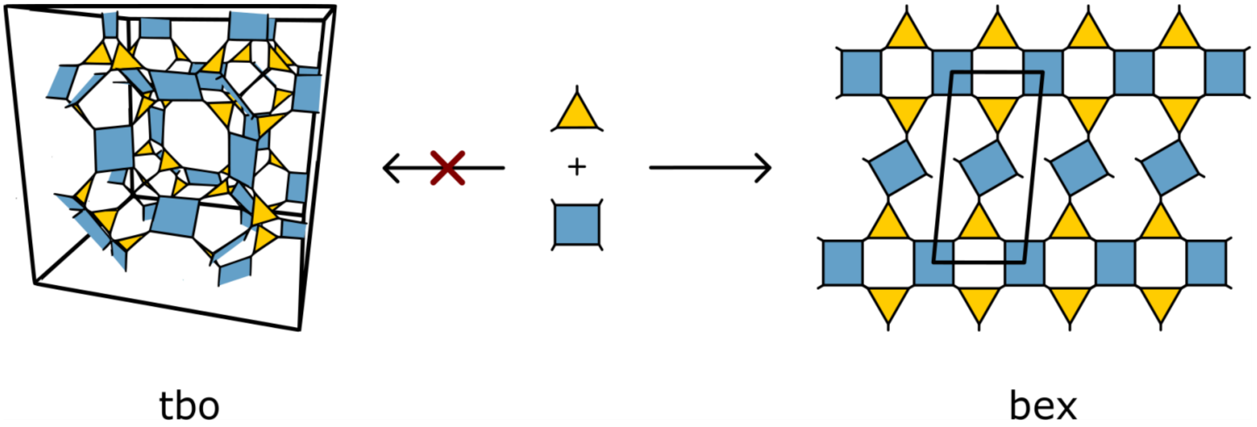

Supplementary Figure 13. Reaction of P and T/Y linkers leads not to the 3D **tbo** net but to the 2D **bex** net.

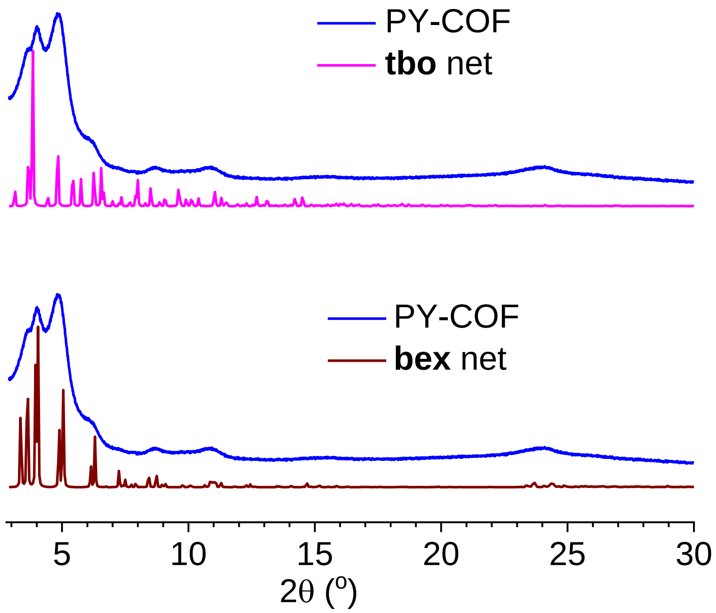

Supplementary Figure 14. Comparison of the experimental PXRD pattern of PY-COF with the simulated patterns for 3D **tbo** and 2D **bex** nets.

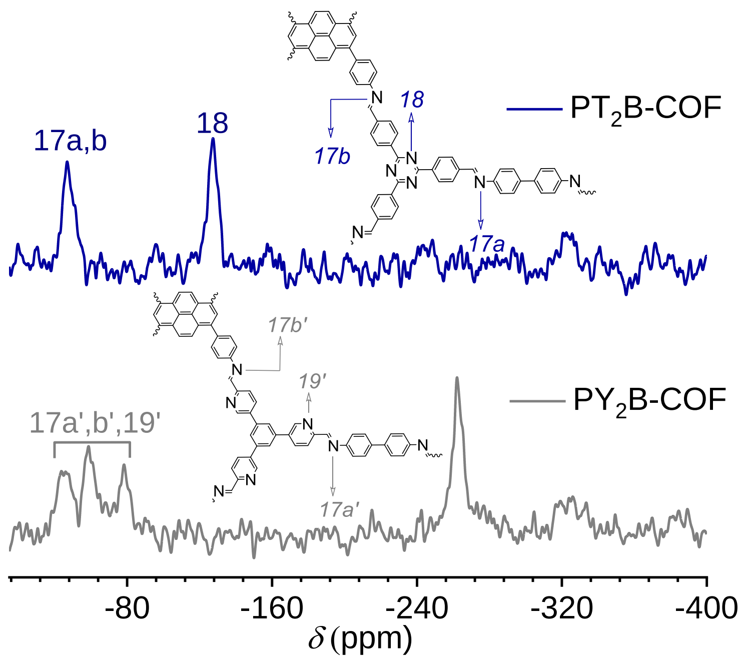

Supplementary Figure 15. ^{15}N ssNMR spectra of PT_2B- and PY_2B-COFs with corresponding assignments.

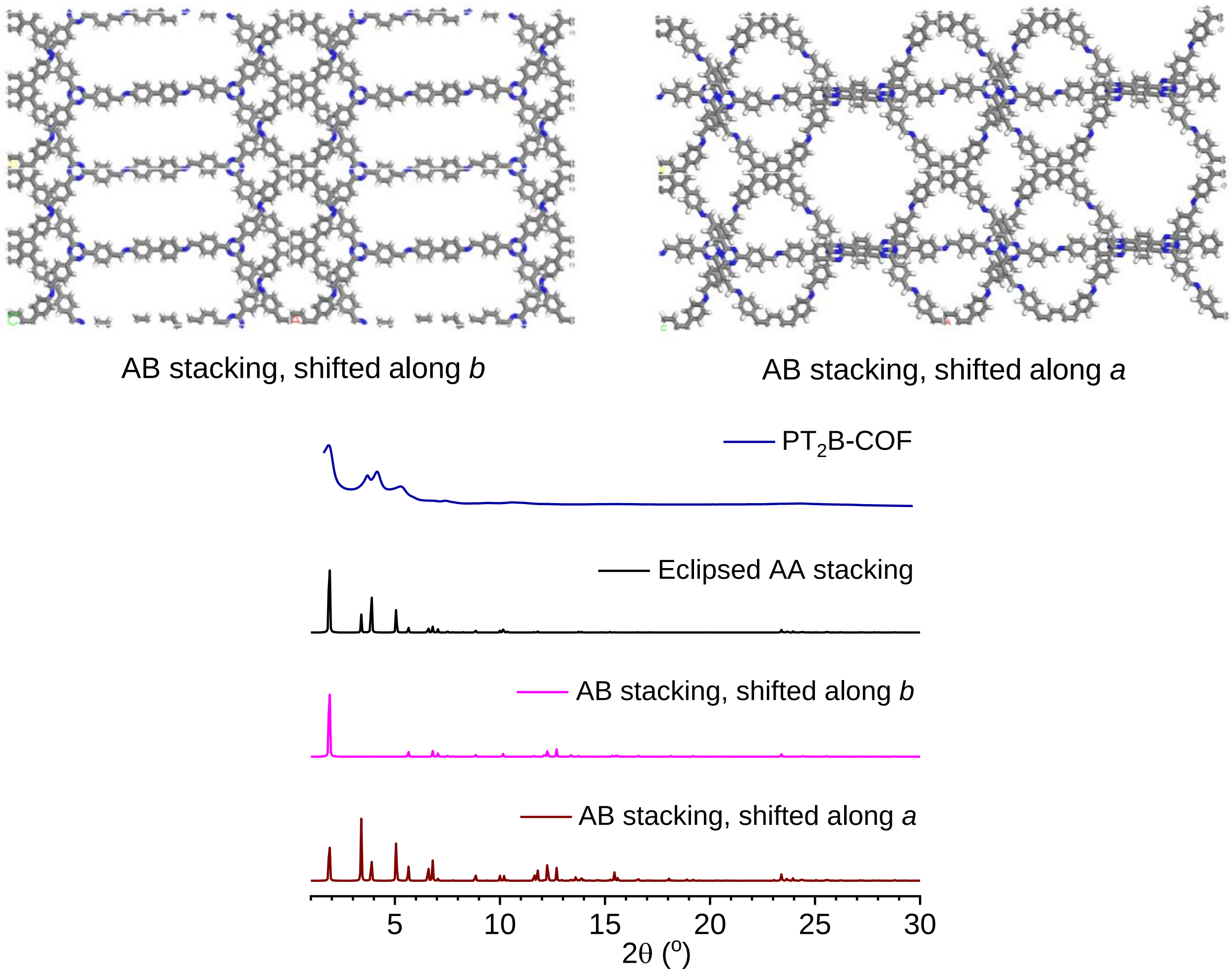

Supplementary Figure 16. Simulated PXRD patterns for AB stacking in PT_2B-COF.

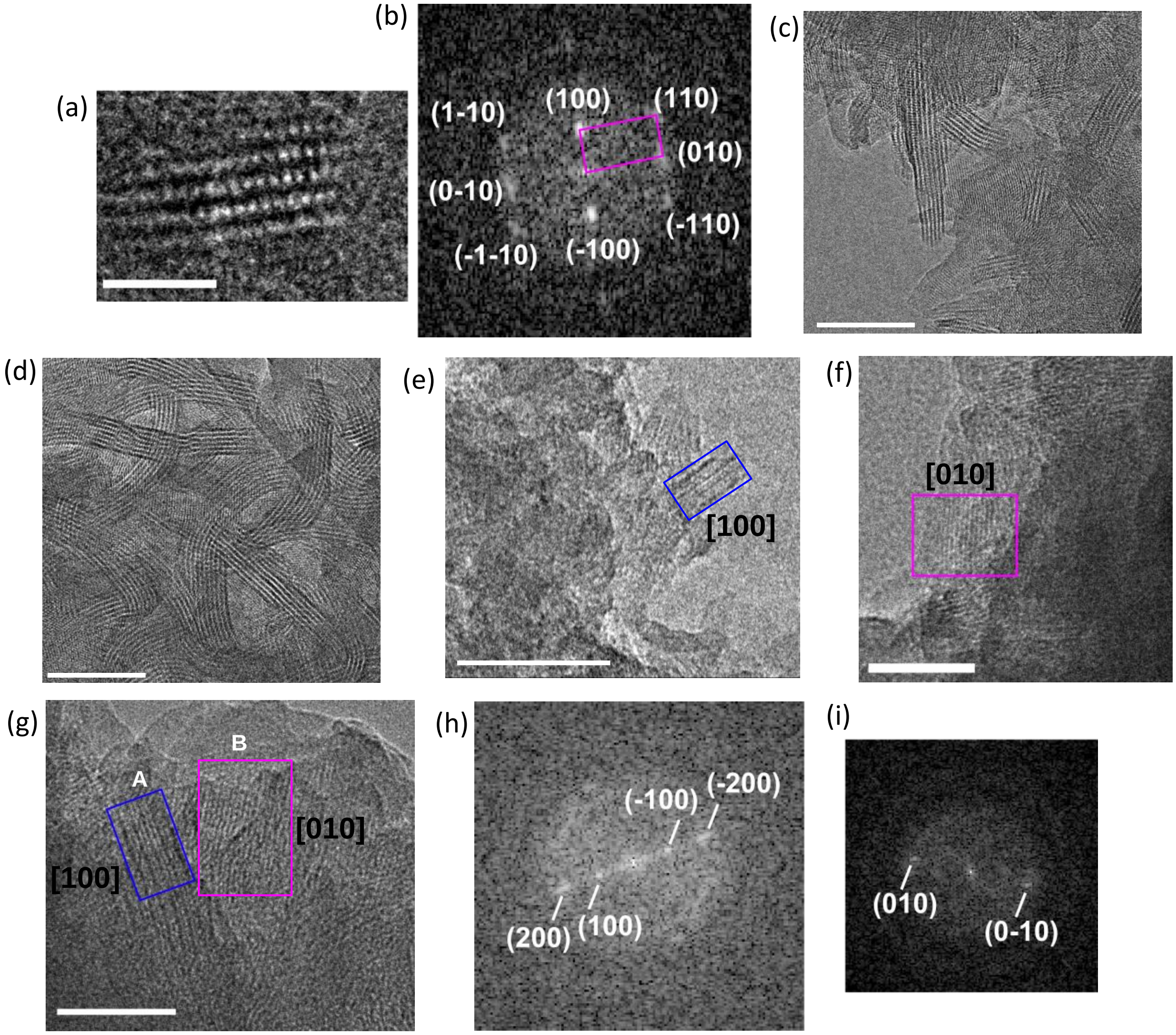

Supplementary Figure 17. (a) A PT_2B-COF crystallite viewed along [001]. (b) Fast Fourier Transform (FFT) filtered image of (a) showing the different planes in the PT_2B-COF crystallite in accordance with the PXRD data. (c) Individual crystallites of PT_2B-COF in different orientations. (d) Elongated strip-like crystallites in PY_2B-COF. (e) TEM image of PY-COF sample. A crystallite showing the lattice spacing in the [100] direction is outlined in blue. (f) A crystallite in the PY-COF sample showing the lattice spacing in the [010] direction is outlined in red. (g) Individual crystallites of PT-COF in different orientations. (h) Fast Fourier Transform (FFT) filtered image of the blue outlined A section in (g) corresponding to the [100] direction. (i) Fast Fourier Transform (FFT) filtered image of the red outlined B section in (g) corresponding to the [010] direction. Scale bars: (a) – 20 nm; (c), (d) – 80 nm; (e) – 100 nm; (f), (g) – 50 nm.

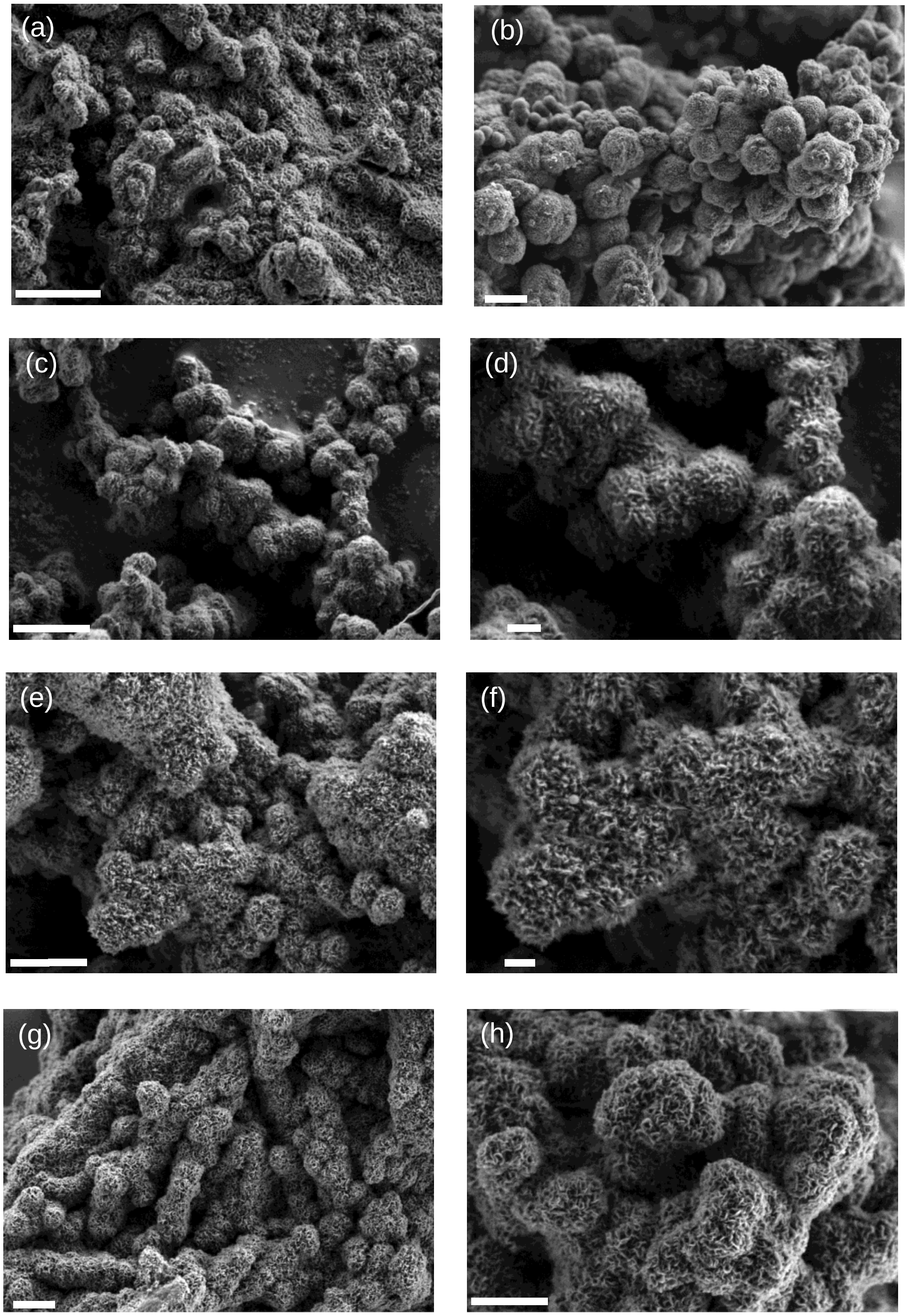

Supplementary Figure 18. SEM images of PT-COF (a,b), PT_2B-COF (c,d), PY-COF (e,f) and PY_2B-COF (g,h). Scale bars: (a) - (c), (e), (g), (h): 2 µm; (d), (f): 400 nm.

Supplementary Table 4. Dimensions of the anisotropic COF crystallites as observed with TEM.

	PT_2B-COF	PY_2B-COF	PT-COF	PY-COF
Crystallite size along *a*	24 - 36 nm (4 - 8 unit cells)	20 - 40 nm (4 - 10 unit cells)	1.3 - 2.6 nm (2 - 4 unit cells)	11 - 16 nm (2 - 3 unit cells)
Crystallite size along *b*	70 - 190 nm (~30 - 80 unit cells)	150 - 250 nm (~70 - 120 unit cells)	4.6 - 8.6 nm (not measurable by TEM)	26 - 65 nm (13 - 33 unit cells)

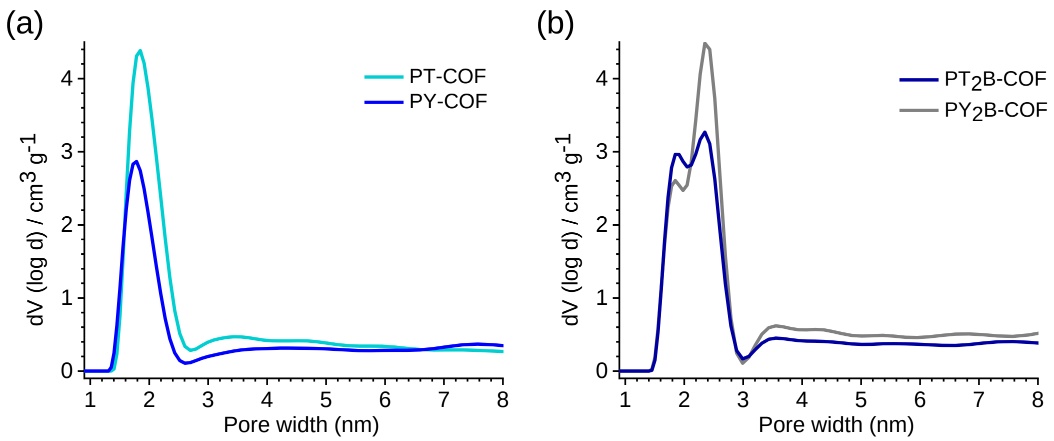

Supplementary Figure 19. Pore size distribution calculated from Ar sorption isotherms.

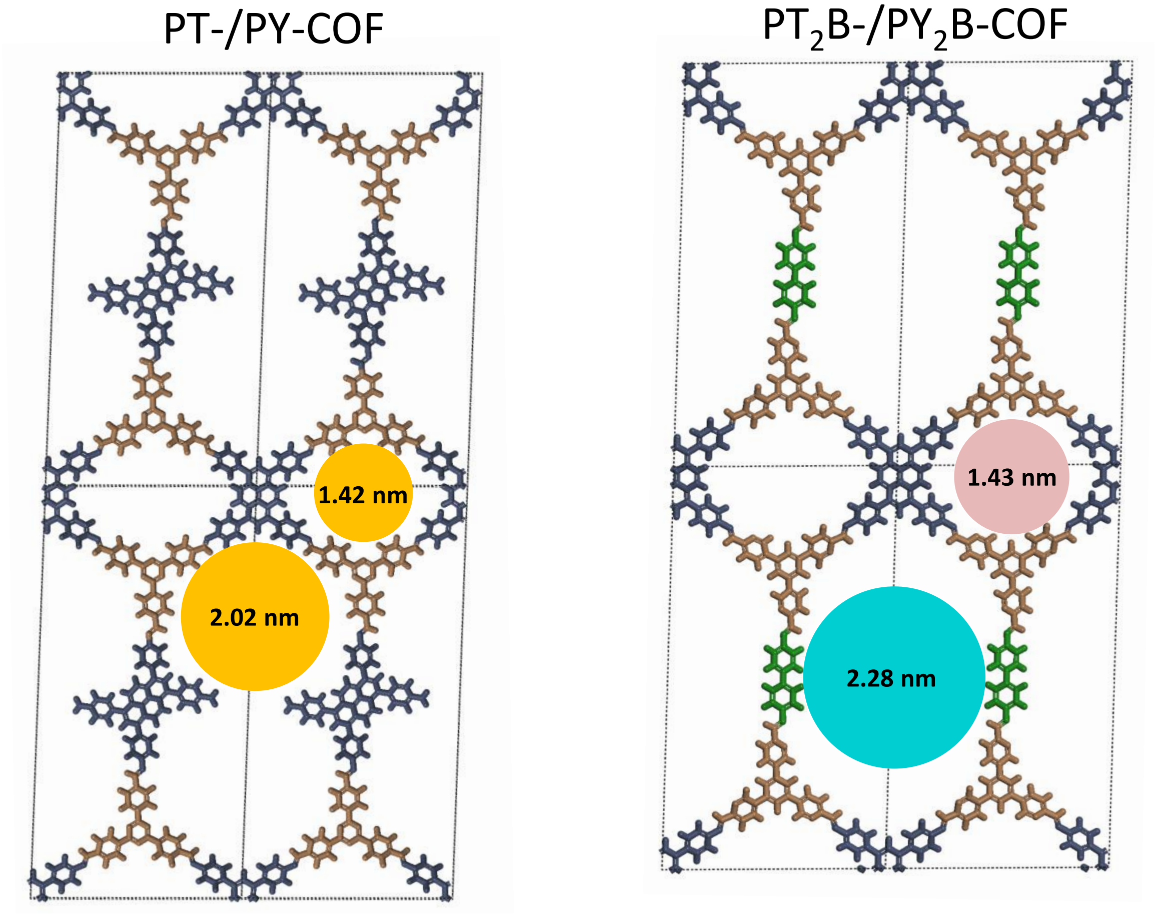

Supplementary Figure 20. Theoretical pore diameters as obtained from the crystal structure model of the COFs. PSD simulations based on sorption isotherms calculated with BIOVIA Materials Studio 2017 (17.1.0.48) also predict pore widths of 1.4 nm and 2.2 nm for PT- and PY-COFs, similar to that obtained from the crystal structure model.

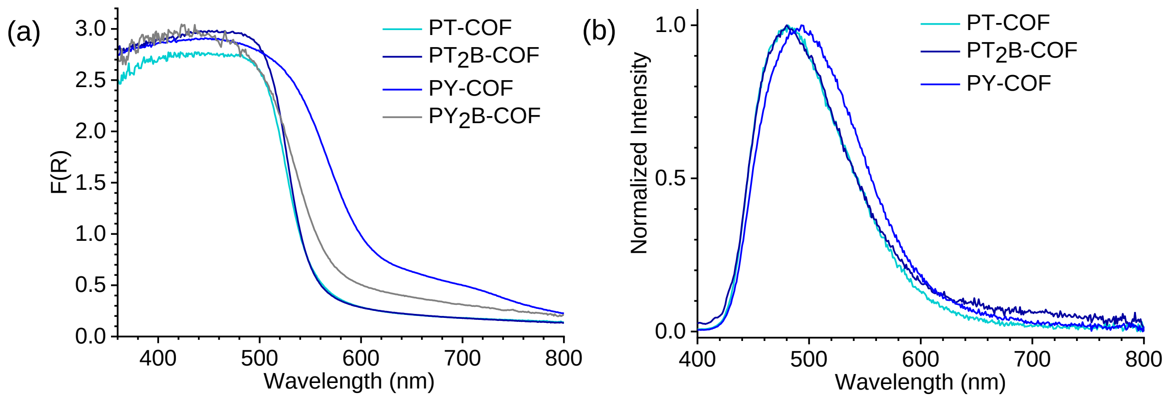

Supplementary Figure 21. (a) Diffuse reflectance spectra of the COFs measured as solids. (b) Photoluminescence spectra of the COFs in acetonitrile dispersion. (λ_{exc} = 380 nm). PY_2B-COF is almost non-emissive and is therefore not shown.

Supplementary Table 5. Photophysical properties of the COFs.

**Sample**	**Band gap, eV**	Φ^a	**τ, ns**	k_r **(× 10^7 s^{-1})^b**	k_{nr} **(× 10^8 s^{-1})^c**
PT-COF	2.24	0.0188	2.55	0.737	3.848
PT_2B-COF	2.24	0.0028	2.74	0.102	3.639
PY-COF	2.05	0.0139	2.75	0.505	3.586
PY_2B-COF	2.18	< 0.001	2.72	< 0.037	> 3.673

Φ - Quantum yield; τ - Fluorscence lifetime; Fluorescence measurements have been done on acetonitrile dispersions. Band gaps have been measured using the Kubelka-Munk function using solid state diffuse reflectance measurements.

k_r - radiative rate: k_{nr} - non-radiative rate; ^{a} λ_{exc}=380 nm; ^{b} $k_r = \Phi/\tau$; ^{c} $k_{nr} = (1-\Phi)/\tau$

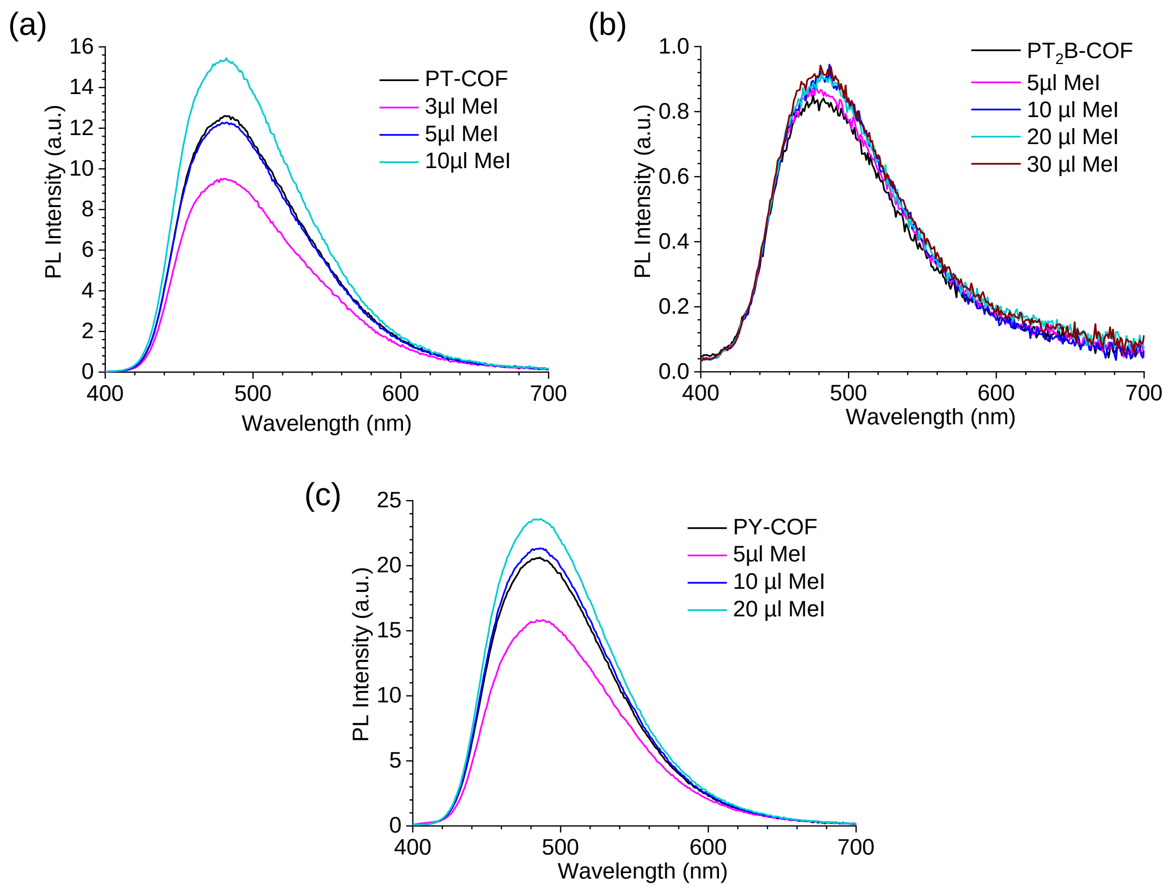

Supplementary Figure 22. Photoinduced electron transfer quenching by the lone pairs on nitrogen atom of the pendant amine groups in PT- and PY-COFs. 2 mg of the respective COF was dispersed in acetonitrile and ~3 equivalents of 2,6-lutidine (per $–NH_2$ group) was added to the dispersion as a base for trapping the HI generated. This mixture corresponds to the COF-only measurement in the figure. After adding MeI, the reaction mixture was allowed to stir for some time at room temperature for the reaction to proceed. PY_2B-COF was too weakly emissive to be studied.

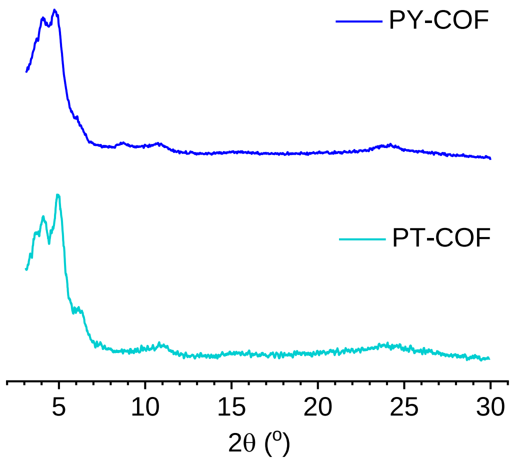

Supplementary Figure 23. PXRD patterns for PT- and PY-COF post photoluminescence studies with MeI in presence of 2,6-lutidine in acetonitrile solvent.

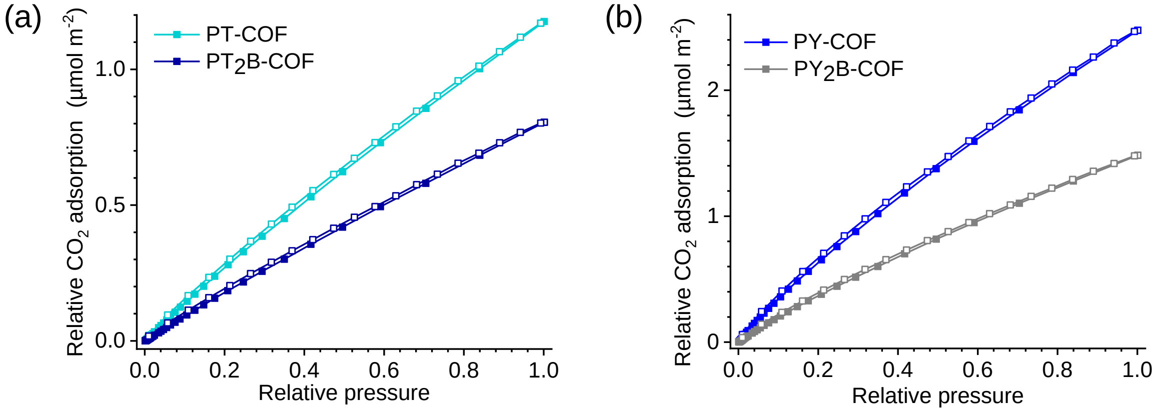

Supplementary Figure 24. CO_2 adsorption isotherms of the COFs at 273 K normalized to the BET surface area of the respective COF samples. Filled and open symbols represent the adsorption and the desorption branches, respectively. Pore size shrinkage is nominal and in the presence of free amine groups, the former should not be the decisive factor towards the higher CO_2 uptake capacity of PT- and PY-COFs.

Supplementary Table 6. Reaction yield and regioselectivity for the COF-catalyzed reaction of 2-hydroxylcinnamaldehyde with trimethylsilyl enol ether.

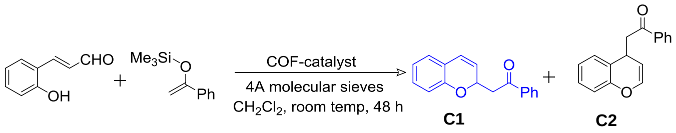

Catalyst	Yield [%][a][b]	Ratio of C1:C2
PY-COF	22	4:1
PT-COF	8	15:1
PY_2B-COF	1-2	-[c]
PT_2B-COF	1-2	-[c]

[a] Detailed reaction conditions in Methods section, [b] Measured using CH_2Br_2 standard, [c] Due to low yields an accurate regioselectivity could not be reported.

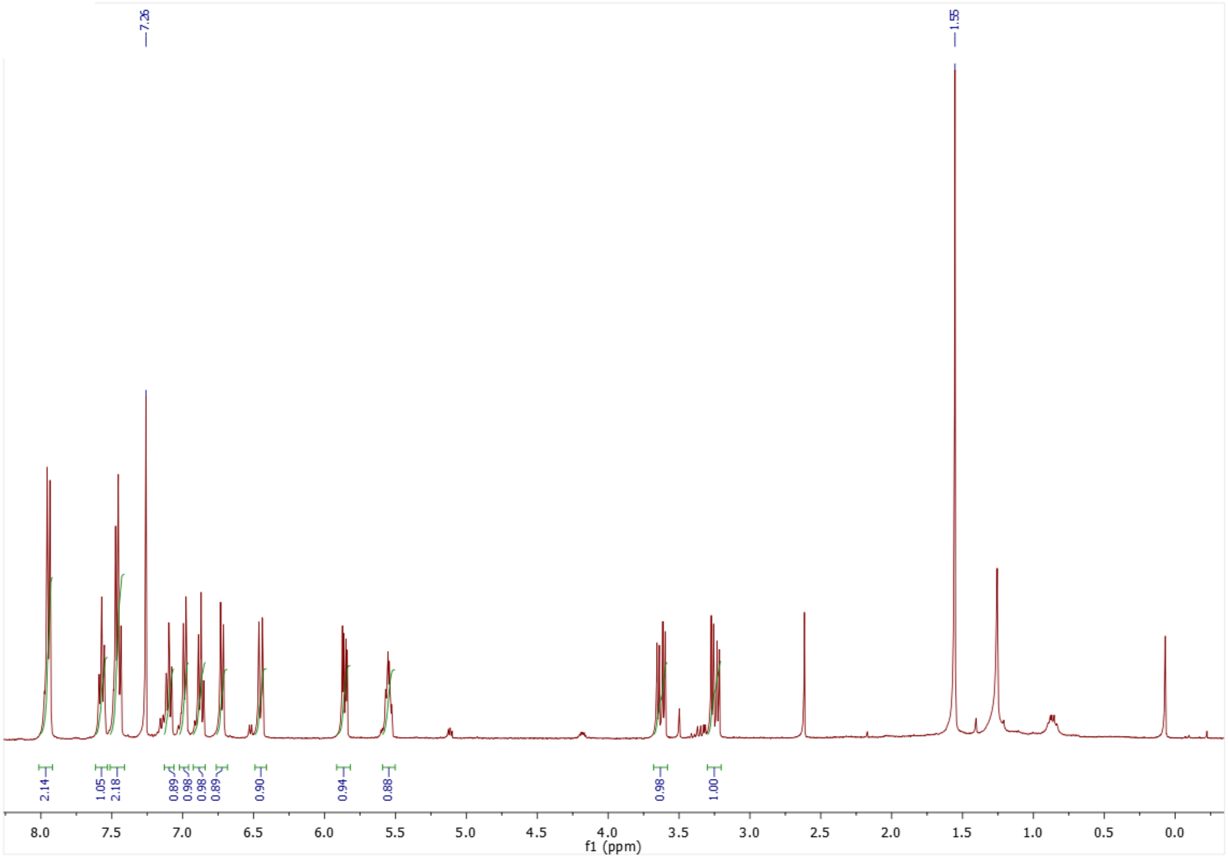

Supplementary Figure 25. ^{1}H NMR spectrum of the isolated chromene C1 from the reaction using benzidine as the aromatic amine catalyst.

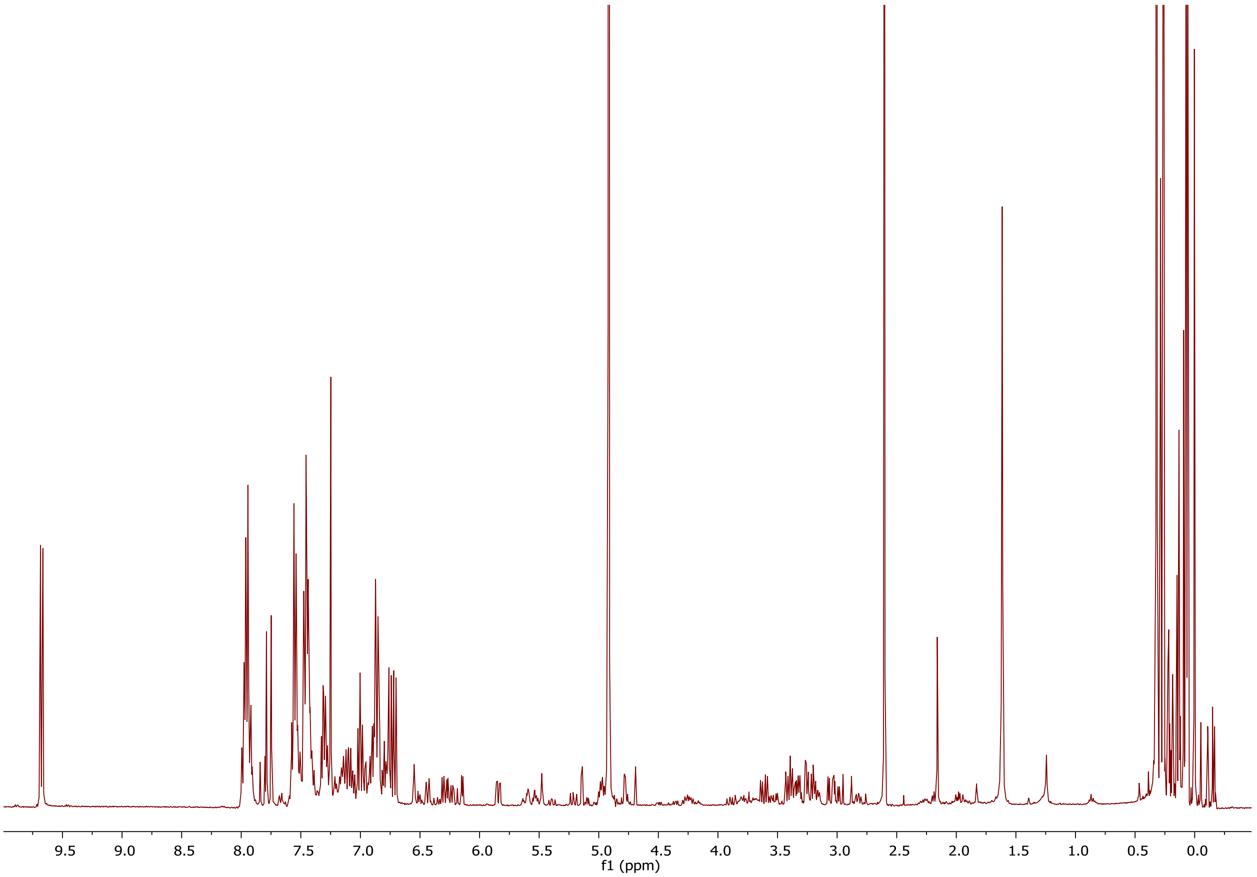

Supplementary Figure 26. ^{1}H NMR spectrum of the crude reaction product using PT-COF as the aromatic amine catalyst. The 1H, dd signal at 5.86 ppm from figure S25 was used as the reference peak for the calculation of reaction yield. The 2H, s signal of CH_2Br_2 is at 4.90 ppm.

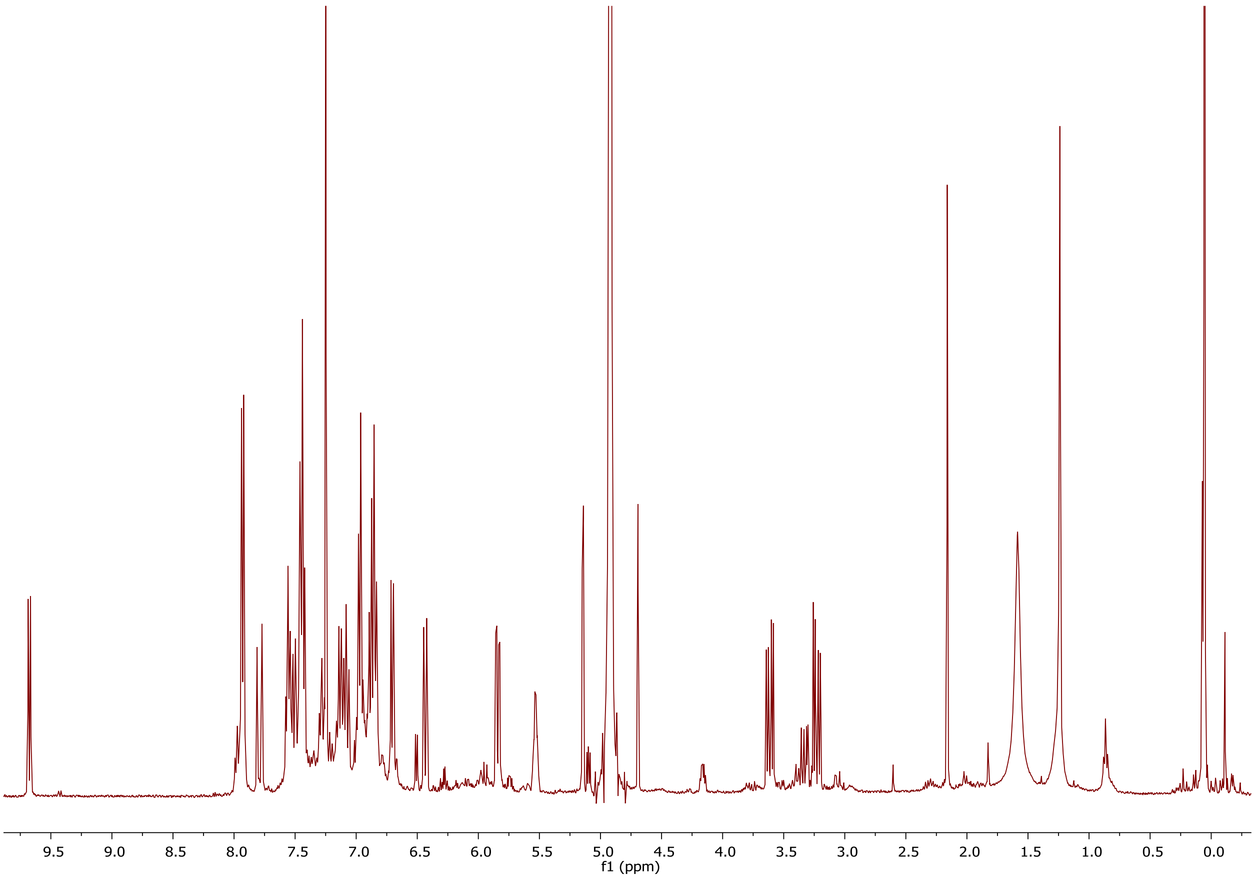

Supplementary Figure 27. ^{1}H NMR spectrum of the crude reaction product using PY-COF as the aromatic amine catalyst. The 1H, dd signal at 3.23 ppm from figure S25 was used as the reference peak for the calculation of reaction yield. The 2H, s signal of CH_2Br_2 is at 4.90 ppm.

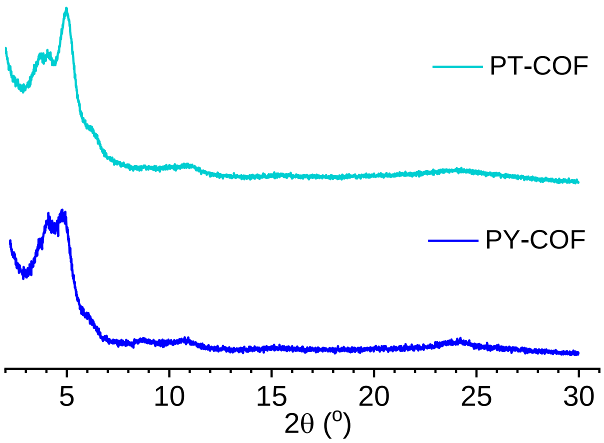

Supplementary Figure 28. PXRD patterns for PT- and PY-COFs after overnight stirring with 2-hydroxycinnamaldehyde and trimethylsilyl enol ether in dichloromethane showing retention of crystallinity. Reactant and solvent stoichiometry were the same as the catalysis reaction. A direct post-catalysis PXRD of the COFs could not be measured because of sample contamination with molecular sieves.

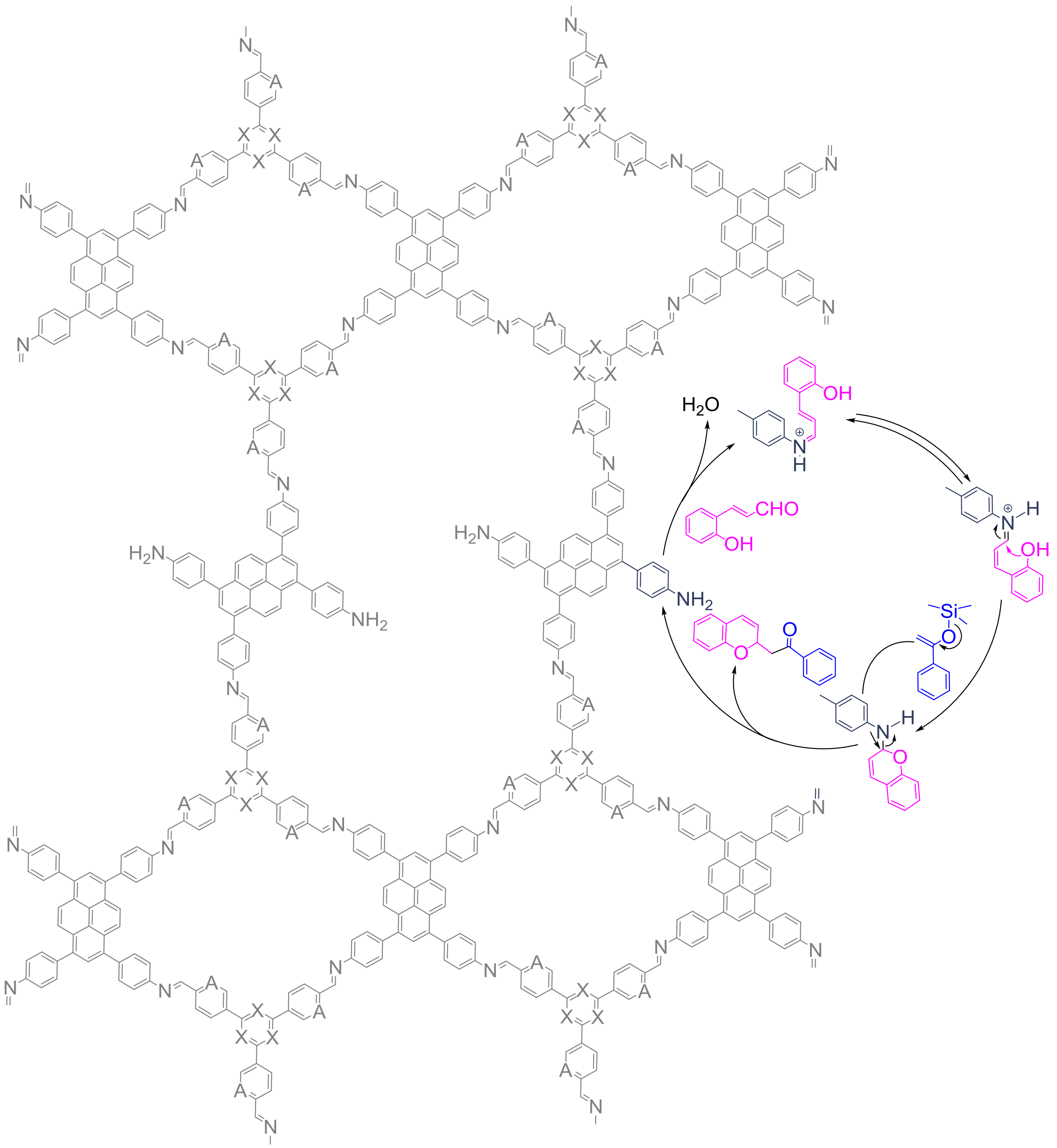

PT-COF: X=N, A=CH
PY-COF: X=CH, A=N

Supplementary Figure 29. Mechanism of the COF-catalyzed reaction between 2-hydroxycinnamaldehyde and trimethylsilyl enol ether as proposed by Yu *et al.*[22]

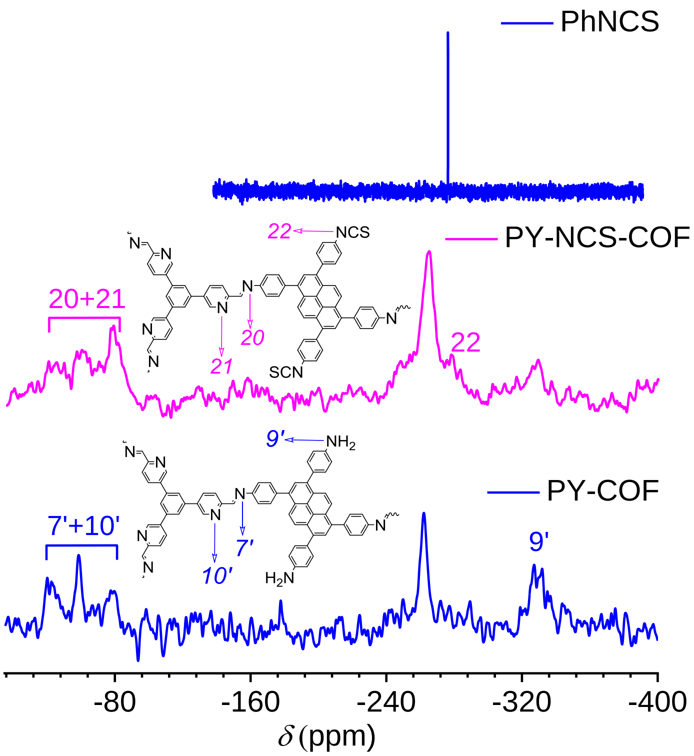

Supplementary Figure 30. ^{15}N ssNMR spectrum of PY-NCS- and PY-COF. The chemical shift of the isothiocyanate nitrogen in PY-NCS-COF matches exactly with the chemical shift of the isothiocyanate nitrogen in phenyl isothiocyanate at -272.1 ppm.

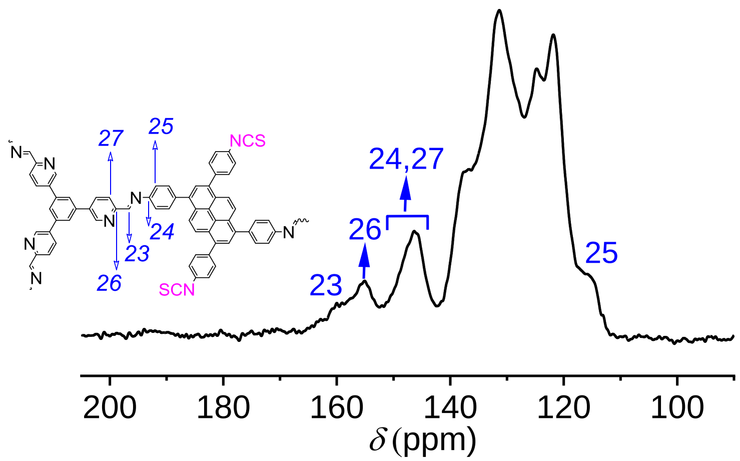

Supplementary Figure 31. ^{13}C ssNMR spectrum of PY-NCS-COF with the corresponding signal assignments. The unassigned signals correspond to the pyrene moiety and the phenyl rings. The isothiocyanate carbon signal cannot be identified (see text for details).

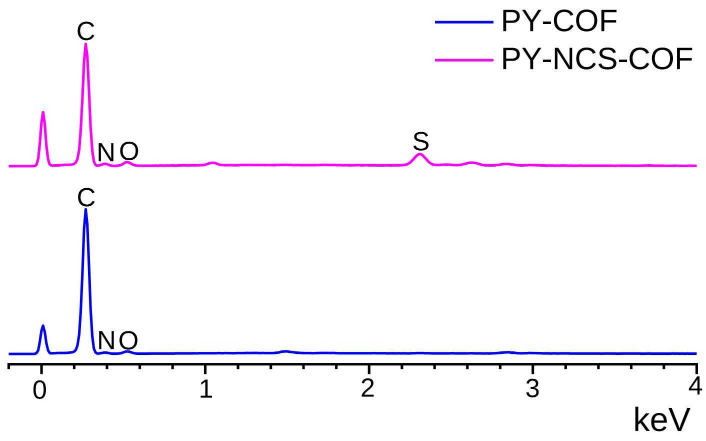

Supplemenatry Figure 32. Energy dispersive X-ray (EDX) spectroscopic analysis of PY- and PY-NCS-COF.

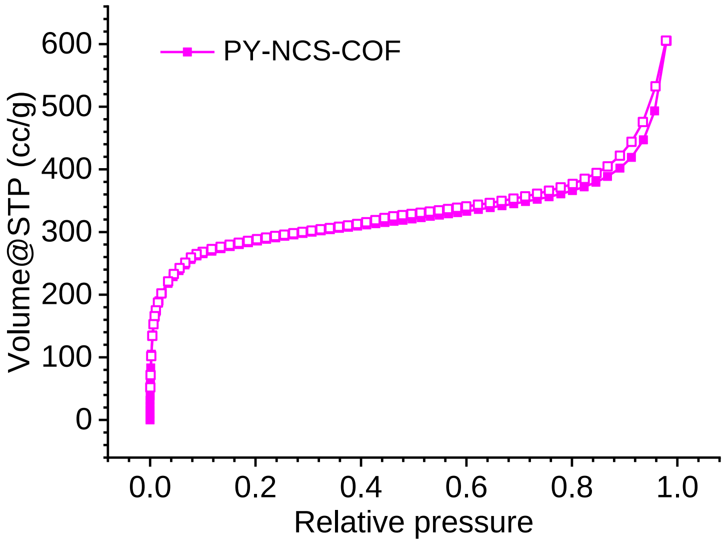

Supplementary Figure 33. Argon sorption isotherm of PY-NCS-COF at 87 K. Filled and open symbols represent the adsorption and the desorption branches, respectively.

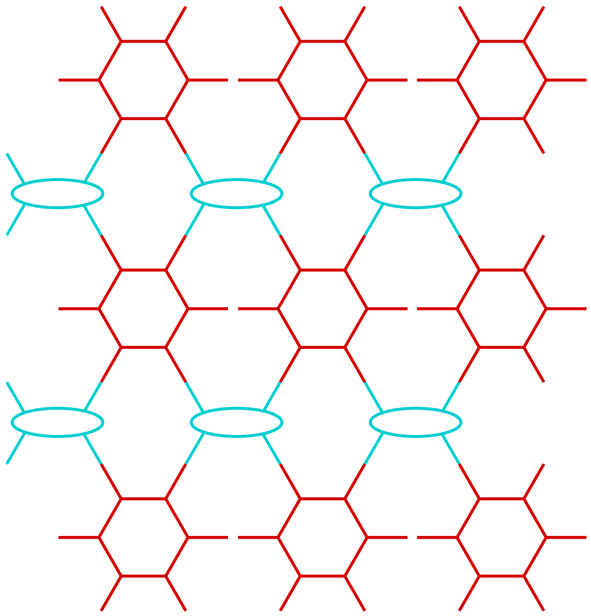

Supplementary Figure 34. Proposed sub-stoichiometric COF composed of appropriate tetra- and hexatopic linkers. Such a structure cannot be derived from a fully condensed [4+6] 2D-COF parent framework.

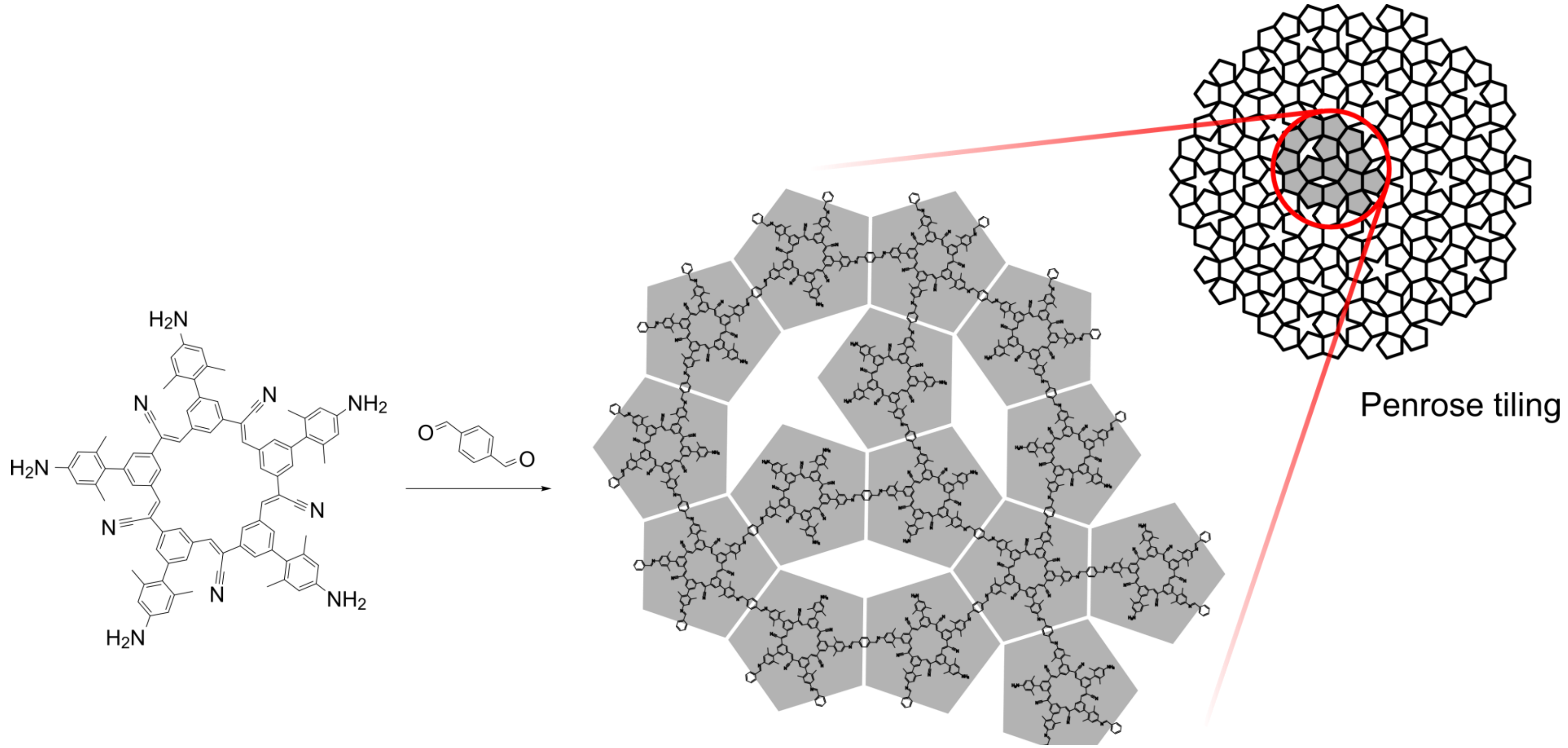

Supplementary Figure 35. Generation of a quasicrystalline COF with a symmetry that relates to the penrose aperiodic tiling based on a pentagonal linker with a cyanostar core.[23] Only through dangling amino groups can a tiling in two dimensions be achieved.

Supplementary References

1. Bao, B., Yuwen, L., Zhan, X. & Wang, L. Water-soluble hyperbranched polyelectrolytes with high fluorescence quantum yield: Facile synthesis and selective chemosensor for Hg^{2+} and Cu^{2+} ions. *J. Polym. Sci., Part A: Polym. Chem.* **48,** 3431- 3439 (2010).

2. Chen, X. *et al.* Towards covalent organic frameworks with predesignable and aligned open docking sites. *Chem.Commun.* **50,** 6161-6163 (2014).

3. Vyas, V. S. *et al.* A tunable azine covalent organic framework platform for visible light-induced hydrogen generation. *Nature Commun.* **6**, 8508 (2015).

4. Rietveld, H. M. A Profile refinement method for nuclear and magnetic structures. *J. Appl. Crystallogr.* **2**, 65-71 (1969).

5. Cheary, R. W., Coelho, A. A., Cline, J. P. Fundamental parameters line profile fitting in laboratory diffractometers. *J. Res. Natl. Inst. Stand. Technol.* **109**, 1-25 (2004).

6. Stephens, P. W. Phenomenological model of anisotropic peak broadening in powder diffraction. *J. Appl. Crystallogr.* **32**, 281-289 (1999).

7. B97-2: P. J. Wilson, T. J. Bradley & D. J. Tozer Hybrid exchange-correlation functional determined from thermochemical data and ab initio potentials. *J. Chem. Phys.* **115,** 9233-9242 (2001).

8. pcsS-2: Jensen, F. Basis set convergence of nuclear magnetic shielding constants calculated by density functional methods. *J. Chem. Theory Comput.* **4,** 719−727 (2008).

9. PBE: Perdew, J. P., Burke, K. & Ernzerhof, M. Generalized Gradient Approximation Made Simple. *Phys. Rev. Lett.* **77,** 3865-3868 (1996).

10. D3: Grimme, S., Antony, J., Ehrlich, S. & Krieg, H. A consistent and accurate ab initio parametrization of density functional dispersion correction (DFT-D) for the 94 elements H-Pu. *J. Chem. Phys.* **132,** 154104 (2010).

11. def2-TZVP: Weigend, F., Häser, M., Patzelt, H. & Ahlrichs, R. RI-MP2: optimized auxiliary basis sets and demonstration of efficiency. *Chem. Phys. Lett.* **294,** 143-152 (1998).

12. Weigend, F. Accurate Coulomb-fitting basis sets for H to Rn. *Phys. Chem. Chem. Phys.* **8,** 1057-1065 (2006).

13. TURBOMOLE, developer version based on version V7.1 2017, a development of University of Karlsruhe and Forschungszentrum Karlsruhe GmbH, 1989-2007, TURBOMOLE GmbH, since 2007; available from http://www.turbomole.com.

14. Ahlrichs, R., Bär, M., Häser, M., Horn, H. & Kölmel, C. Electronic structure calculations on workstation computers: The program system turbomole. *Chem. Phys. Lett.* **162,** 165–169 (1989).

15. Kussmann, J. & Ochsenfeld, C. Pre-selective screening for matrix elements in linear-scaling exact exchange calculations. *J. Chem. Phys.* **138,** 134114 (2013).

16. Kussmann, J. & Ochsenfeld, C. Preselective screening for linear-scaling exact exchange-gradient calculations for graphics processing units and general strong-scaling massively parallel calculations. *J. Chem. Theory Comput.* **11,** 918-922 (2015).

17. de Mello, J. C., Wittmann, H. F. & Friend, R. H. An improved experimental determination of external photoluminescence quantum efficiency. *Adv. Mater.* **9,** 230-232 (1997).

18. Thommes, M. *et al.* Physisorption of gases, with special reference to the evaluation of surface area and pore size distribution (IUPAC Technical Report). *Pure Appl. Chem*. **87,** 1051–1069 (2015).

19. Li, X., *et al.* Facile transformation of imine covalent organic frameworks into ultrastable crystalline porous aromatic frameworks. *Nature Commun.* **9**, 2998 (2018).

20. Waller, P. J. *et al.* Chemical conversion of linkages in covalent organic frameworks. *J. Am. Chem. Soc.* **138,** 15519−15522 (2016).

21. Han, X. Chiral covalent organic frameworks with high chemical stability for heterogeneous asymmetric catalysis. *J. Am. Chem. Soc.* **139,** 8693−8697 (2017).

22. Yu, C. *et al.* Aniline-promoted cyclization–replacement cascade reactions of 2-hydroxycinnamaldehydes with various carbonic nucleophiles through in situ formed N,O-acetals. *Chem. Eur. J.* **22,** 9240 – 9246 (2016).

23. Lee, S., Chen, C.-H. & Flood, A. H. A pentagonal cyanostar macrocycle with cyanostilbene CH donors binds anions and forms dialkylphosphate [3]rotaxanes. *Nature Chem.* **5**, 704–710 (2013).
