# Supplementary material for: Sub-stoichiometric 2D covalent organic frameworks from tri- and tetratopic linkers
Source: Nat Commun. 2019 Jun 19;10:2689. doi: 10.1038/s41467-019-10574-6 (PMC6584614; doi:10.1038/s41467-019-10574-6)
Supplement: Supplementary file 2 — Description of Additional Supplementary Files [file 41467_2019_10574_MOESM2_ESM.pdf]

Description of Additional Supplementary Files

File name: Supplementary Data 1

Description: Rietveld refined structure model of PT-COF

File name: Supplementary Data 2

Description: Rietveld refined structure model of PY-COF

File name: Supplementary Data 3

Description: Rietveld refined structure model of PT_2B-COF

File name: Supplementary Data 4

Description: Rietveld refined structure model of PY_2B-COF

File name: Supplementary Data 5

Description: Rietveld refined structure model of PY-NCS-COF
